# Evaluating the effectiveness of conservation measures for European grassland‐breeding waders

**DOI:** 10.1002/ece3.4532

**Published:** 2018-10-09

**Authors:** Samantha E. Franks, Maja Roodbergen, Wolf Teunissen, Anne Carrington Cotton, James W. Pearce‐Higgins

**Affiliations:** ^1^ British Trust for Ornithology Thetford UK; ^2^ Sovon Dutch Centre for Field Ornithology Nijmegen The Netherlands; ^3^ British Trust for Ornithology Scotland Stirling UK; ^4^ Conservation Science Group, Department of Zoology University of Cambridge Cambridge UK

**Keywords:** agricultural intensification, agri‐environment schemes, conservation, farmland, interventions, meadow birds, population declines, reproductive success, shorebirds, site protection

## Abstract

Farmland birds are among the most threatened bird species in Europe, largely as a result of agricultural intensification which has driven widespread biodiversity losses. Breeding waders associated with grassland and arable habitats are particularly vulnerable and a frequent focus of agri‐environment schemes (AES) designed to halt and reverse population declines. We review existing literature, providing a quantitative assessment of the effectiveness of policy and management interventions used throughout Europe to improve population and demographic metrics of grassland‐breeding waders. Targeted AES and site protection measures were more likely to be effective than less targeted AES and were ten times more likely to be effective than would be expected by chance, particularly for population trend and productivity metrics. Positive effects of AES and site protection did not appear synergistic. Management interventions which had the greatest chance of increasing population growth or productivity included modification of mowing regimes, increasing wet conditions, and the use of nest protection. Success rates varied according to the species and metric being evaluated. None of the policy or management interventions we evaluated were associated with a significant risk of negative impacts on breeding waders. Our findings support the use of agri‐environment schemes, site protection, and management measures for grassland‐breeding wader conservation in Europe. Due to publication bias, our findings are most applicable to intensively managed agricultural landscapes. More studies are needed to identify measures that increase chick survival. Despite broadly effective conservation measures already in use, grassland‐breeding waders in Europe continue to decline. More research is needed to improve the likelihood and magnitude of positive outcomes, coupled with wider implementation of effective measures to substantially increase favorable land management for these species.

## 1 INTRODUCTION

Farmland birds are declining across Europe (BirdLife International 2015; Donald, Green, & Heath, 2001), a pattern matched by other aspects of biodiversity (Pe'er et al., 2014) leading to long‐term declines in once common and dominant species across the continent (Inger et al., 2015). Declines have primarily been attributed to changes in land management, including both intensification of agriculture and land abandonment (Chamberlain, Fuller, Bunce, Duckworth, & Shrubb, 2000; Donald, Sanderson, Burfield, & van Bommel, 2006), as well as to associated practices such as the application of pesticides and herbicides, changes in cropping patterns and type, and natural system modifications including hydrological changes to favor agricultural production (Chamberlain & Fuller, 2000; Flohre et al., 2011). This combination of factors has reduced ecological heterogeneity and also food resources for wildlife at critical times of the year (Benton, Vickery, & Wilson, 2003; Burns et al., 2016; Chamberlain et al., 2000).

Agricultural intensification was largely driven by the EU's Common Agricultural Policy (CAP). The ensuing negative impacts of intensification on biodiversity have led to several major CAP reforms and the development of agri‐environment schemes (AES) which pay farmers for land management that benefits biodiversity; however, solutions to halt or reverse biodiversity declines have thus far proved inadequate (Donald, Pisano, Rayment, & Pain, 2002; Donald et al., 2006; Pe'er et al., 2014).

Throughout Europe, birds associated with grasslands and agricultural habitats comprise the highest proportion of threatened species (23%; BirdLife International 2015). Grassland‐breeding waders are especially sensitive and include Eurasian oystercatcher *Haematopus ostralegus*, northern lapwing *Vanellus vanellus*, black‐tailed godwit *Limosa limosa*, Eurasian curlew *Numenius arquata*, common redshank *Tringa totanus*, ruff *Calidris pugnax*, the Baltic‐breeding population of dunlin *Calidris alpina schinzii*, and common snipe *Gallinago gallinago*. Since the early 1980s, these species have shown rapid population declines throughout Europe (European Bird Census Council 2015): four are classified as “vulnerable” (oystercatcher, lapwing, redshank, and curlew) and two as endangered (black‐tailed godwit and ruff) on the red list of the EU27 (BirdLife International 2015), four (oystercatcher, lapwing, black‐tailed godwit, and curlew) are listed as “near threatened” on the global IUCN red list (IUCN 2015), while Baltic dunlin is one of the most vulnerable wader populations in Europe (Thorup, 2006). Despite these conservation concerns, only ruff and Baltic dunlin feature on the EU Birds Directive Annex l list of threatened species, while all except dunlin can be hunted under certain restrictions. None are priorities for funding under the EU's LIFE program (European Commission 2014).

A likely demographic driver of population declines is low productivity (Roodbergen, van der Werf, & Hoetker, 2012) due to a combination of: (a) earlier cropping, mowing, and grazing dates with agricultural intensification and climate change (Kleijn et al., 2010) resulting in destruction of eggs and chicks by agricultural machinery and livestock (e.g., Kruk, Noordervliet, & terKeurs, 1997); (b) reduced food quality and/or availability in intensively managed grassland monocultures or as a result of large‐scale drainage, resulting in poorer chick growth and/or survival (Kentie, Hooijmeijer, Trimbos, Groen, & Piersma, 2013; Schekkerman & Beintema, 2007); and (c) increased predation of eggs and chicks due to high predator densities, combined with greater susceptibility to predation in degraded breeding habitat (Bolton, Tyler, Smith, & Bamford, 2007; Kentie, Both, Hooijmeijer, & Piersma, 2015; Roos, Smart, Gibbons, & Wilson, 2018; Schekkerman, Teunissen, & Oosterveld, 2009; Teunissen, Schekkerman, Willems, & Majoor, 2008).

Considerable efforts have been made in some countries to conserve grassland‐breeding birds (Kleijn & Sutherland, 2003). Although some local projects have been successful (e.g., Peach, Lovett, Wotton, & Jeffs, 2001; Perkins, Maggs, Watson, & Wilson, 2011), declines continue at a national and European scale. Numerous studies have evaluated the success of conservation measures at various scales (e.g., Breeuwer et al., 2009; Kleijn, Berendse, Smit, & Gilissen, 2001; Kleijn & Sutherland, 2003; O'Brien & Wilson, 2011; Smart et al., 2014; Walker et al., 2018), but Europe‐wide evaluations of both AES and underlying conservation management measures are largely lacking.

Large‐scale conservation action requires: (a) the effective use of policy instruments to facilitate positive change and (b) the adoption of meaningful management interventions (Vickery & Tayleur, 2018). Within Europe, the EU Birds Directive, Common Agricultural Policy, and associated national legislation provide key policy mechanisms to support the establishment of Special Protected Areas (SPAs) and agri‐environment schemes for bird conservation, while a range of different management interventions may be deployed at sites to address threats or facilitate population recovery. Here, we review and quantitatively assess the effectiveness of both policy and management interventions that have been used throughout Europe in an attempt to improve breeding conditions for grassland‐breeding waders and to halt declines and/or restore populations. By quantitatively assessing these broad policy mechanisms, we provide much‐needed evidence about their overall effectiveness. This is particularly relevant at a time of significant political change in the UK and ongoing policy reform in Europe. By evaluating the effectiveness of more specific management interventions, we test the extent to which particular measures are more or less likely to be successful, and under which circumstances, in order to inform future management prescriptions for grassland‐breeding waders.

## 2 METHODS

### 2.1 Literature review

A systematic review of the primary scientific literature was conducted using ISI Web of Knowledge. Keyword search and logic terms were selected to identify studies on the relevant species dealing with conservation management approaches and outcomes (Supporting Information Appendix S1). This search generated 4,897 results which were then screened by title, abstract, and content (SF and AC), resulting in a final set of 58 studies and 481 records (lines of data). Qualifying studies had to evaluate the impact of management interventions on the relevant species during the breeding season in Europe, in relation to measures of abundance, occupancy, changes in these metrics, survival, or reproductive success. In addition to the primary scientific literature, MR identified 16 relevant gray literature studies which contributed a further 107 records (Supporting Information Appendix S2).

### 2.2 Data extraction and synthesis

SF (primary literature) and MR (gray literature) constructed the analytical dataset, extracting data for each study. Each record in the dataset included study “meta‐data” information (e.g., location) and variables to be included in subsequent analyses, including analytical information from each study (e.g., sample size), the species evaluated, and key data on the range of interventions evaluated and their effect on the population and/or demographic metric(s) measured (Supporting Information Appendix S3; Table S2). Where a study tested the effect of interventions on multiple metrics and/or species simultaneously, we extracted each metric and species combination as a separate record (Supporting Information Appendix S3). We excluded records for ruff from the analysis as the number of records (2) was too small for meaningful inclusion.

#### 2.2.1 Interventions

We simplified our assessment of interventions by categorizing them into eight broad classes (Supporting Information Table S2): two address policy mechanisms: AES and site protection; six test management interventions: mowing, grazing, agrochemicals (fertilizer, herbicides, or pesticides), water management (both groundwater and surface water), nest protection (either from agricultural activities or predation; Supporting Information Appendix S3), predator control. Management measures are often used individually or in combination and may form the basis by which AES are implemented or protected areas are managed (Supporting Information Figure S1). For this reason, policy and management interventions are analyzed separately.

While interventions on the ground may be heterogeneous in their approaches (Supporting Information Table S2), they are simplified for the purposes of our analysis. Agri‐environment schemes and their component management measures can vary substantially by country, so we simplified our further classification of scheme type into two broad categories: “higher‐level” schemes available in certain countries (e.g., UK, the Netherlands) targeted at achieving outcomes for waders specifically, and “basic” schemes, which included generic “biodiversity‐friendly” interventions not targeted at particular species. For simplicity and to deal with model convergence problems, we did not distinguish between different types of site protection, such as local nature reserves, national (e.g., Site of Special Scientific Interest in the UK), or international (e.g., Natura 2000) designations. However, we acknowledge that there may, in some cases, be an association between certain forms of site protection and the use or prohibition of particular management interventions. For each intervention, we determined whether it was (a) not evaluated by the study; (b) applied, if the study evaluated the effect of applying or increasing the intervention above the baseline reference level; or (c) reduced, if the study evaluated the effect of removing or decreasing the intervention below the baseline reference level (Supporting Information Table S2). SF and MR cross‐checked a subset of records to ensure consistency in classification.

#### 2.2.2 Population and demographic metrics

Studies evaluated the effects of one or more interventions on: abundance, abundance change, occupancy, occupancy change, adult survival, or productivity (nest survival, chick survival, fledglings per pair, and recruitment). For simplicity and to resolve model convergence problems, we pooled metrics into three categories: count (abundance/occupancy), trend (abundance/occupancy change), and productivity (nest/chick survival and fledglings per pair). There were too few studies evaluating survival or recruitment for these metrics to be included in the analysis. Because of likely spatial bias in the deployment of interventions, such that they are targeted at wader “hotspots” of occurrence and/or abundance (Kleijn & Sutherland, 2003), we tested for interactions between intervention success and metric category. We specifically focused on intervention success in relation to trend and productivity metrics when interpreting the results. These metrics are more likely to reflect the effectiveness of interventions, as opposed to count metrics, which may be particularly affected by interventions being targeted toward locations with high densities of breeding waders.

#### 2.2.3 Effect size

Where possible, we extracted the effect size of the metric in response to interventions (Supporting Information Appendix S3). However, due to having a limited number of suitable studies in each intervention category and a lack of common reporting metrics across studies, we cannot provide a formal estimate of pooled effect size using meta‐analysis approaches which incorporate uncertainty and study sample size and biases (Koricheva, Gurevitch, & Mengersen, 2013). Instead, to inform the reader about the potential magnitude of effect associated with different interventions, we provide the effect size range for each intervention (Supporting Information Table S3).

### 2.3 Statistical analysis

We used the probability of intervention success or failure as the main metric to assess the effectiveness of interventions. This was modeled using generalized linear mixed‐effects models (GLMMs) with a binomial error distribution and a logit link function (Supporting Information Table S4). All models were fitted using *glmer* in the lme4 package in R (Bates et al., 2014; R Core Team 2015) unless specified otherwise.

In three separate analyses, we modeled success rate for (a) individual interventions overall (both policy and management interventions); (b) variation in individual interventions between species and metric categories; and (c) interventions applied in combination. Because of its low frequency of occurrence, failure rate was modeled only for individual interventions overall.

#### 2.3.1 Response variables

For the analysis of intervention success, we classified an intervention outcome as successful if there was a statistically significant, positive impact on a metric (1), or as unsuccessful if there was a non‐significant or significant, negative impact (0, Supporting Information Table S2). For the analysis of intervention failure, we classified failures as significant, negative outcomes (1), in contrast to non‐significant or positive impact interventions (0). We considered an intervention to have a measurable impact if the predicted probability of success (success rate) or failure (failure rate) across studies differed significantly from the expectation that the same outcome could occur by chance, based on the *p* = 0.05 threshold for significance of the underlying studies. If the modeled 95% confidence intervals on the success (or failure) rate did not overlap 5%, this indicated an intervention that succeeded (or failed) more often than expected by chance, which we regarded as an indicator of effectiveness.

#### 2.3.2 Evaluating the importance of confounding covariates

The probability of success or failure could vary with potentially confounding covariates which we were not expressly interested in: study duration, the analytical approach used, sample size, literature type, study quality, and metric bias (Supporting Information Table S2). Hence, we first modeled whether any of these covariates affected success rate. Literature type was the only significant covariate when applying single‐term deletion and likelihood ratio tests to a global GLMM of potential confounding variables (Supporting Information Table S5). Primary literature studies had a lower success rate than gray literature studies. However, including literature type as a fixed effect in subsequent models created convergence problems, so we included study as a random intercept term in all models to account for at least some of the variance attributable to literature type.

##### Evaluating overall success and failure rates of individual interventions

We first modeled overall success rate of individual interventions in nine separate models (Analysis 1a, Supporting Information Table S4). For each intervention, we filtered the data to use only those records where the focal intervention was evaluated. If the filtered dataset included only records with a single level of the intervention (e.g., only records where the intervention was applied and none where it was reduced), success (*π*_*i*_) was fitted in an intercept‐only model with both study (*a*_1_) and species (*a*_2_) as random intercepts:

$$\text{logit}\left(\pi_{i}\right)=\alpha +a_{1i}+a_{2i}+\varepsilon_{i}$$

If the dataset included two levels of the intervention (e.g., grazing applied and reduced), success was fitted against the intervention covariate:

$$\text{logit}\left(\pi_{i}\right)=\alpha +\beta_{1}\times {\text{Intervention}}_{i}+a_{1i}+a_{2i}+\varepsilon_{i}$$

We modeled overall failure rates of individual interventions in eight separate models as above (Analysis 1b; Supporting Information Table S4; we were unable to model the probability of failure of predator control due to unsolvable convergence issues).

##### Evaluating species‐ and metric‐specific responses

Next, we modeled individual intervention success as above, but added either a species (Analysis 2a) or a metric (Analysis 2b) covariate to each of the nine models to examine differences in success between species and the population or demographic metric evaluated (Supporting Information Table S4). Models were fitted as either:

$$\text{logit}\left(\pi_{i}\right)=\alpha +\beta_{1}\times {\left(\text{Species or Metric}\right)}_{i}+a_{1i}+\varepsilon_{i}$$

for interventions with only a single level, or as:

$$\text{logit}\left(\pi_{i}\right)=\alpha +\beta_{1}\times {\left(\text{Species or Metric}\right)}_{i}+\beta_{2}\times {\text{Intervention}}_{i}+\beta_{3}\times {\left(\text{Species or Metric}\right)}_{i}*{\text{Intervention}}_{i}+a_{1i}+\varepsilon_{i}$$

for interventions with two levels. To solve problems with model convergence as a result of complete or quasi‐complete separation and/or singularities, we (a) refitted some models with a reduced dataset to exclude categories with too few observations and (b) applied a Bayesian framework to model fitting using *bglmer* in the blme package, which applies a weak prior to the fixed effect parameters (Supporting Information Table S4; Dorie, 2015).

There were insufficient failures to examine variation in the likelihood of failed interventions between species or metrics. Due to the limited sample size, we were also unable to examine whether metric‐specific responses varied between species.

##### Evaluating interventions applied in combination

While the above analyses assessed the effectiveness of interventions individually, in many cases, the focal intervention is applied alongside others, which may result in an overestimate of the effectiveness of the focal intervention if (a) success rate is attributable to the combined suite or to one of the other interventions in the suite and (b) a study did not control for the combined application of interventions using a multivariate framework. Consequently, we also modeled intervention effectiveness in combination (Analysis 3a). We explicitly sought to examine the combined effectiveness of policy mechanisms separately from management interventions, because the two are intrinsically linked (AES success will be a function of the managements adopted), and because some studies evaluated policy measures without considering the underlying management interventions.

First, we examined the success rate of AES and site protection in combination, while controlling for species as a fixed covariate and study (*a*_1_) as a random intercept. We filtered the dataset to include only records where these interventions were evaluated and then combined the three different possible combinations of AES and site protection as levels of a single categorical variable (Policy Mechanism variable levels: AES + no site protection; no AES + site protection; AES + site protection) and modeled success rate as:

$$\text{logit}\left(\pi_{i}\right)=\alpha +\beta_{1}\times \text{Policy Mechanism combination}+\beta_{2}\times {\text{Species}}_{i}+a_{1i}+\varepsilon_{i}$$

Secondly, we modeled the success rate of management interventions applied in combination (Analysis 3b) as:

$$\text{logit}\left(\pi_{i}\right)=\alpha +\beta_{1}\times {\text{mowing}}_{i}+\beta_{2}\times {\text{grazing}}_{i}+\beta_{3}\times {\text{agrochemicals}}_{i}+\beta_{4}\times {\text{water}}_{i}+\beta_{5}\times \text{nest}\;{\text{protection}}_{i}+\beta_{6}\times \text{predator}\;{\text{control}}_{i}+\beta_{7}\times {\text{Species}}_{i}+a_{1i}+\varepsilon_{i}$$

where the significance of each intervention was tested using single‐term deletion and likelihood ratio tests. We were unable to model the interactive effects between interventions and species, or between interventions and metric category, due to sample size limitations. We ranked unique intervention combinations according to their model‐predicted probability of success to evaluate whether using more interventions in combination tended to result in higher success rates.

## 3 RESULTS

### 3.1 Literature review summary

Published studies were heavily biased toward the UK and the Netherlands (45% and 26% of studies, respectively, see Supporting Information Figure S2). Agri‐environment schemes were the most studied intervention (42% of studies, Supporting Information Figure S3), while lapwing was the most commonly studied species (73% of studies, Supporting Information Figure S4). Productivity was the most frequently evaluated metric in relation to conservation interventions (50% of studies, Supporting Information Figure S5), closely followed by abundance and occupancy (49%) and abundance and occupancy change (30%).

#### 3.1.1 Evaluating overall success and failure rates of individual interventions

**Figure 1:**
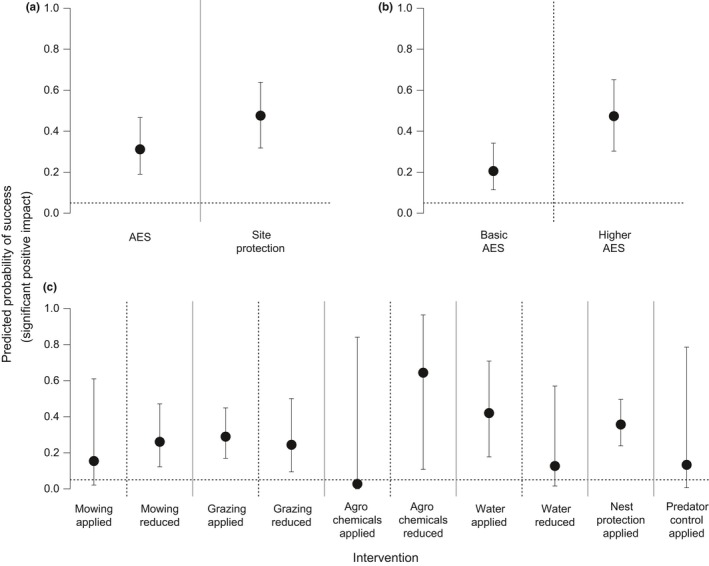
The predicted probability (mean ± 95% confidence interval) that an intervention will result in a successful outcome for (a) AES and site protection; (b) basic‐ vs. higher‐level AES; and (c) management interventions. The dotted horizontal line represents the threshold at which we would expect success by random chance, at a significance level of *p* = 0.05. Solid vertical lines separate probabilities predicted by different models, while dotted vertical lines separate intervention levels

The predicted probability of success of AES (Figure 1a) and site protection were six and ten times more likely than expected by chance to be associated with a positive outcome for breeding waders. Higher‐level AES (Figure 1b) had a higher probability of success than basic‐level AES. Apart from applying mowing, applying agrochemicals, reducing wet conditions, and applying predator control, most management interventions were found to be associated with a fourfold to eightfold greater probability of a successful outcome than expected by chance (Figure 1c, Supporting Information Table S6). Applying mowing or grazing and reducing wet conditions tended to have the highest probabilities of failure, though in no cases were these significantly greater than expected by chance (Supporting Information Figure S6, Table S6).

#### 3.1.2 Evaluating species‐ and metric‐specific responses

**Figure 2:**
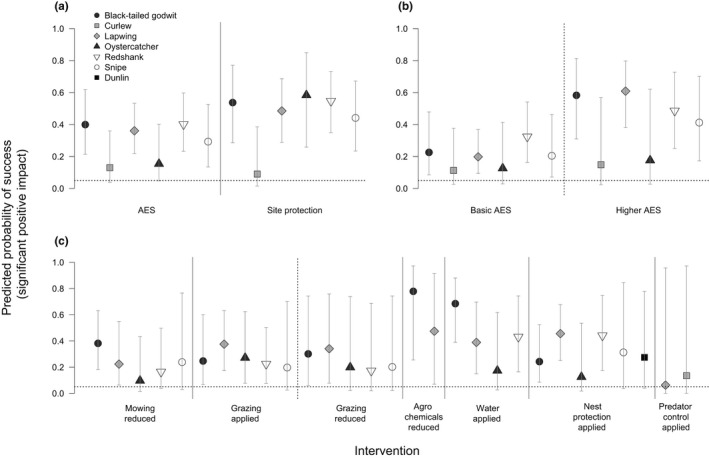
The species‐specific predicted probability of success (mean ± 95% confidence interval) of (a) AES and site protection; (b) basic‐ vs. higher‐level AES; and (c) management interventions. The dotted horizontal line represents the 5% threshold for success as expected by random chance. Solid vertical lines separate probabilities predicted by different models, while dotted vertical lines separate intervention levels. Species were not evaluated for an intervention if sample sizes were insufficient (generally <5 records)

Agri‐environment schemes, particularly higher‐level AES, were most likely to be successful for black‐tailed godwit, lapwing, redshank, and snipe (Figure 2a,b, Supporting Information Table S6). Only for oystercatcher and curlew did AES fail to increase the probability of success from random. Site protection was likely to be effective for all species apart from curlew. Reduced mowing was most likely to succeed for black‐tailed godwit and lapwing (Figure 2c, Supporting Information Table S6). Applying grazing was most likely to be successful for black‐tailed godwit, lapwing, oystercatcher, and redshank, while both reduced grazing and reduced agrochemical use were most likely to succeed for black‐tailed godwit and lapwing. Both nest protection and increasing wet conditions were most likely to be successful for black‐tailed godwit, lapwing, and redshank. Applying predator control did not result in a greater likelihood of success than expected by chance for either curlew or lapwing, the only two species evaluated.

**Figure 3:**
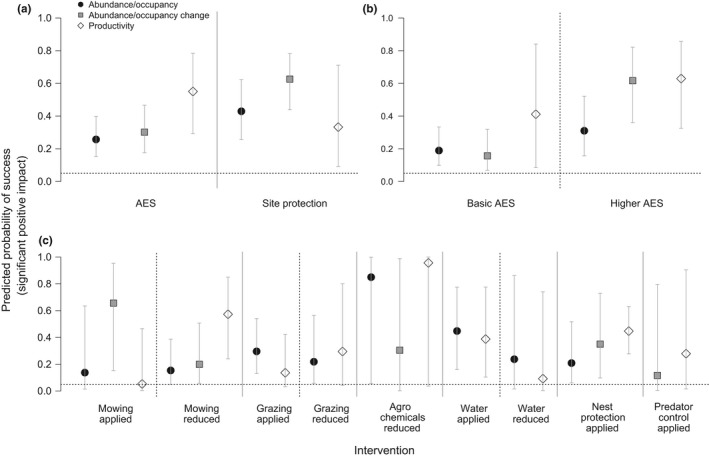
The metric‐specific predicted probability of success (mean ± 95% confidence interval) of (a) AES and site protection; (b) basic‐ vs. higher‐level AES; and (c) management interventions. The dotted horizontal line represents the 5% threshold for success as expected by random chance. Solid vertical lines separate probabilities predicted by different models, while dotted vertical lines separate intervention levels. A metric was not evaluated for an intervention if its sample size was insufficient (generally <5 records)

When evaluating the probability of intervention success according to metric, wader productivity was most likely to respond positively to AES, with a greater than 50% probability of a positive impact. Wader population change was most likely to respond positively to site protection, with a 60% chance of positive abundance or occupancy change through time. Wader population trends and productivity were most likely to respond positively to higher‐level AES interventions, and positive outcomes tended to be more likely for both of these metrics under higher‐level AES as compared to basic‐level AES (Figure 3a,b, Supporting Information Table S6).

When evaluating management interventions, applying mowing was most likely to positively impact trend metrics (Figure 3c, Supporting Information Table S6), while reductions in mowing were most likely to increase productivity, in both cases with a more than 50% mean probability of success. The effects of manipulating grazing were most apparent when evaluating variation in count metrics, but with a lower rate of success. While reducing the input of agrochemicals had a high probability of increasing both count and productivity metrics, there was a high level of uncertainty associated with this intervention. Increasing wet conditions was associated with significantly more positive count and productivity metrics about 40% of the time, while nest protection was most likely to positively influence both productivity and trend metrics with a similar frequency of success. Applying predator control did not result in a greater likelihood of success than expected by chance for either trend or productivity metrics.

#### 3.1.3 Evaluating interventions applied in combination

**Figure 4:**
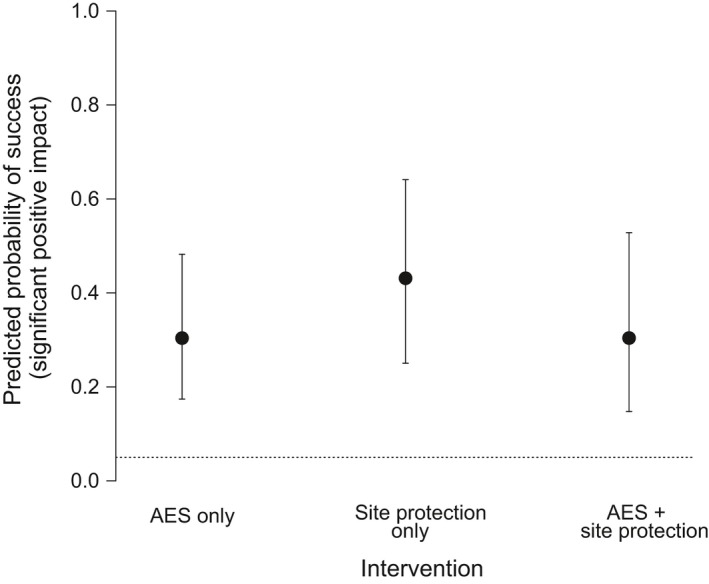
The predicted probability of success (mean ± 95% confidence interval) for AES and site protection alone, as well as combined. The dotted horizontal line represents the 5% threshold for success as expected by random chance

**Figure 5:**
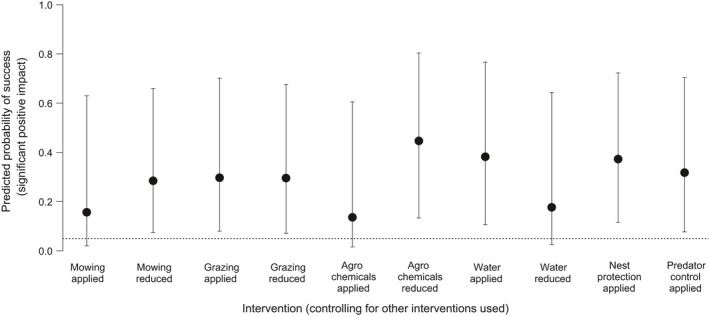
The predicted probability of success (mean ± 95% confidence interval) of different management interventions, controlling for the use of multiple interventions in combination. The dotted horizontal line represents the 5% threshold for success as expected by random chance

There was no evidence that AES and site protection in combination provided any greater likelihood of success over AES (Tukey contrast, *p* = 1.0) or site protection (*p* = 0.31) alone (Figure 4). We found no indication that success rate depends on one single management intervention when multiple interventions were applied in combination (Supporting Information Table S7), and the probability of success did not appear to be related to the number of interventions used in combination (Supporting Information Figure S7). While the uncertainty of the likelihood of success increased for all interventions in comparison with Analysis 1, only the effectiveness of predator control was notably affected when controlling for other interventions, with a greater likelihood of success than expected by chance, in contrast to Analysis 1 (Figure 5, Supporting Information Table S6).

## 4 DISCUSSION

Our assessment of policy mechanisms and management interventions aimed at conserving European grassland‐breeding waders found evidence supporting the positive impacts of agri‐environment schemes and site protection, as well as a range of broad management interventions. Although the literature was largely biased toward studies in Western Europe, our review nevertheless provides one of the most comprehensive assessments of the effectiveness of wader conservation measures in European intensive agricultural landscapes to date, and importantly indicates that interventions as currently applied are unlikely to negatively impact breeding wader communities.

### 4.1 The success of policy interventions

Current evidence supporting the positive conservation impacts of AES is mixed (e.g., Perkins et al., 2011; Baker, Freeman, Grice, & Siriwardena, 2012; but see Verhulst, Kleijn, & Berendse, 2007; Davey et al., 2010), with broader assessments of these schemes delivering largely equivocal results (e.g., Batary, Dicks, Kleijn, & Sutherland, 2015; Kleijn & Sutherland, 2003; Scheper et al., 2013). Although our analyses of overall probability of success and species‐level success are potentially confounded by schemes being targeted at areas with already high wader densities, we explicitly test for differences between count, trend, and productivity metrics to reduce the likely impact of this “spatial bias” problem on our inferences, with trend and productivity metrics more likely to reflect genuine biological responses to interventions. Our review identifies positive outcomes for wader population trends and productivity associated with the use of AES, especially the use of targeted schemes, providing evidence that the Europe‐wide investment in AES (Batary et al., 2015) can indeed benefit breeding waders.

Our findings also support the effectiveness of site protection, for example, through the EU's Natura 2000 network (Donald et al., 2007; Pe'er et al., 2014), with positive outcomes being especially frequent for wader population trends. Although we did not identify a synergistic or additive effect of both AES and site protection in our analysis, there is evidence that this can happen in some circumstances (Smart et al., 2014).

While AES and site protection are therefore likely to be effective, broad policy tools for wader conservation, the extent of their success at improving breeding conditions will depend on underlying management interventions, the range of species, and habitat conditions at a site, and the scale at which these tools are applied. Protected sites with multiple breeding species will likely require a range of management measures targeted toward the differing requirements of individual species; similarly, the effectiveness of AES will depend on applying schemes at a sufficiently broad scale and including a range of prescriptions which can be suitably tailored to local conditions. As such, a “one‐size‐fits‐all” approach to either policy tool is unlikely to provide the conservation benefit that could be achieved through a more flexible, outcomes‐driven, evidence‐based approach (e.g., Perkins et al., 2011).

### 4.2 The success of management interventions

Broadly speaking, our assessment indicates that conservation management can produce positive impacts for breeding waders. However, the most effective use of particular interventions requires considering their specific timing, duration, and intensity and the characteristics of the particular site or habitat. For example, due to contrasting habitat requirements, intervention responses may vary between species breeding at the same site (e.g., Buchanan, Pearce‐Higgins, Douglas, & Grant, 2017) and may have contrasting impacts on occupancy and abundance, productivity, and survival. Our assessment provides only a broad evaluation of the overall patterns, whereas responses are likely to vary with local context.

#### 4.2.1 Mowing and grazing

Early and frequent mowing in intensively farmed areas can be damaging for nests and young chicks, or can reduce the subsequent availability of invertebrates for surviving chicks (Kentie et al., 2013; Schekkerman et al., 2009). In other cases, mowing tall swards can create or maintain appropriate open habitat and a short sward, particularly in extensively farmed/abandoned areas (Devereux, Mckeever, Benton, & Whittingham, 2004; Vickery et al., 2001). Our results indicate that mowing as a conservation tool requires careful consideration. While mowing can benefit abundance and occupancy trends, for example, by creating suitable sward structure and potentially attracting breeding birds into an area, reductions in frequency or delays in the timing of mowing have a high likelihood of enhancing wader productivity. Therefore, when applied as a conservation measure, mowing during the breeding season should be avoided as it is likely to destroy nests or chicks, or reduce food resources for chicks.

Our findings suggest that grazing benefits several wader species, likely by creating and maintaining a more heterogeneous and less dense sward structure and composition preferred for nesting and foraging (Norris et al., 1998; Sharps, Garbutt, Hiddink, Smart, & Skov, 2016; Verhulst, Kleijn, Loonen, Berendse, & Smit, 2011; Żmihorski, Pärt, Gustafson, & Berg, 2016). However, particular habitat requirements are likely to be species‐specific, possibly requiring an experimental approach to identify the most beneficial grazing strategy (Durant, Tichit, Kerneis, & Fritz, 2008). Furthermore, inherent risks to grazing management need careful consideration when determining the appropriate stocking density and timing and duration of grazing. Although our results are marginal as to whether reducing grazing is likely to enhance productivity, previous work suggests that even light grazing may lead to significant nest mortality through trampling, depending upon the livestock involved, or alter vegetation structure thereby increasing nest predation (Hart, Milsom, Baxter, Kelly, & Parkin, 2002; Mandema, Tinbergen, Ens, & Bakker, 2013; Pakanen, Luukkonen, & Koivula, 2011; Sharps, Smart, Skov, Garbutt, & Hiddink, 2015; Sharps et al., 2016). Our results support this potential for negative effects, as there is a trend toward applied grazing leading to an increased likelihood of management failure. Potential solutions for reducing nest mortality while maintaining appropriate habitat include grazing prior to and/or after the main nesting period, employing rotational grazing through time or across different compartments, or further reducing stocking density below currently recommended levels (Pakanen, Aikio, Luukkonen, & Koivula, 2016; Sharps et al., 2016). Ideally, designing optimal management requires information to model the potentially complex population and demographic impacts of different grazing regimes (e.g., Sabatier, Doyen, & Tichit, 2010).

#### 4.2.2 Agrochemicals

Organic farming has previously been shown to increase breeding wader abundance (Henderson et al., 2012; Piha, Tiainen, Holopainen, & Vepsalainen, 2007), and our findings suggest that reductions in agrochemical use are broadly associated with higher wader abundance and occupancy. While few studies have closely investigated the potential mechanisms responsible for this effect, reduced agrochemical use may increase invertebrate abundance (Boatman et al., 2004), which has declined considerably over the past 30 years (Hallmann et al, 2017), and/or increase production of a more herb‐rich, less dense sward (Kentie et al., 2013). Both factors are likely to enhance productivity, though our results only suggest a trend in this direction.

#### 4.2.3 Wet conditions

Improving wet conditions by raising water levels, reducing drainage, or using scrapes and foot drains to create open water, can be an important determinant of occupancy and nesting density at the start of the breeding season (Eglington et al., 2008; Smart, Gill, Sutherland, & Watkinson, 2006). Furthermore, wet conditions can provide critical foraging habitat with high food availability for both adults and chicks, particularly later in the breeding season when the water table is lower (Eglington et al., 2010; Kahlert, Clausen, Hounisen, & Petersen, 2007). Our results indicate that improved wet conditions can support greater numbers and higher occupancy of waders and can also benefit productivity, though certain species may respond more favorably than others, which should be considered when manipulating water levels at sites with multiple breeding species.

#### 4.2.4 Nest protection and predator control

Low productivity as a consequence of nest and chick predation and destruction by agricultural activities is likely a key factor limiting European wader populations (MacDonald & Bolton, 2008; Roodbergen et al., 2012). Nest protection and predator control may both reduce nest and chick loss, but can differ in their appropriateness and success depending on the specific management context. Our results indicate nest protection can benefit productivity and also abundance/occupancy trends, but effectiveness may be species‐dependent. Nest protection requires careful consideration of potential trade‐offs and should also be combined with habitat improvement for chicks to avoid creating an ecological trap (Kentie et al., 2013). Leaving unmown patches around or using markers at individual nests may reduce agricultural nest loss, but increase vulnerability to predation (Kragten, Nagel, & De Snoo, 2008; Kentie et al., 2015; but see Zámečník, Kubelka, & Šálek, 2018). While effective at increasing nest survival (Pauliny, Larsson, & Blomqvist, 2008; Smith, Pullin, Stewart, & Sutherland, 2011), nest cages are unlikely to benefit chick survival and may potentially increase predation risk of incubating adults or result in nest abandonment (Isaksson, Wallander, & Larsson, 2007). Fencing is also effective at increasing nest survival (Malpas et al., 2013; Smith et al., 2011), and for large fenced areas may additionally enhance chick survival (Rickenbach et al., 2011). Fences may encourage higher settlement densities due to predator exclusion, as suggested by observed positive effects on abundance/occupancy trends, which can consequently improve group defense against avian predators (Berg, Lindberg, & Kallebrink, 1992). While both can have significant positive effects on productivity, cages and fencing have high maintenance costs and any successful predator incursions may be costly.

An alternative approach to nest protection is control of generalist predators, including foxes, corvids, and mustelids (Bolton et al., 2007; Fletcher, Aebischer, Baines, Foster, & Hoodless, 2010). When considered alone, the success rate of predator control was highly variable and unlikely to benefit productivity more than expected by chance, although it was found to be more successful when controlling for the combined application of other interventions. While previously reviewed as generally effective at increasing productivity and population size (Côté & Sutherland, 1997; Smith, Pullin, Stewart, & Sutherland, 2010), the success of predator control for waders may depend on the specific predators, their spatial and temporal abundance, the effectiveness of different control measures, the sustained use of control measures over time (Bodey, Mcdonald, Sheldon, & Bearhop, 2011; Bolton et al., 2007), as well as the suite of other interventions being used, which may account for the variable responses among the studies conducted.

### 4.3 Study limitations

Our findings are heavily biased toward Western European intensive pastoral and arable landscapes. In the more extensively managed grasslands of Central and Eastern Europe, different levels and types of management may be needed to increase heterogeneity and improve habitat suitability (Żmihorski, Kotowska, Berg, & Pärt, 2016). For example, mowing and grazing may be critical for maintaining open landscapes and preventing secondary succession in abandoned grasslands (Baldi, Batary, & Erdos, 2005). While our findings are therefore likely to be most relevant in the largely intensive agricultural landscapes of Western Europe, our assessment of the effectiveness of policy and management measures can also likely be extrapolated to areas of Central and Eastern Europe experiencing farmland bird declines as a consequence of agricultural intensification (e.g., Reif & Vermouzek, 2018).

Although we accounted for a study bias toward lapwing by including species as a random effect or covariate where possible, and by considering species‐specific variation in intervention success, a limited sample size reduced our ability to examine the effectiveness of particular interventions for certain species. Furthermore, relatively few studies examined the effect of interventions on chick survival; thus, while measures may be effective at increasing nest success, evidence for an overall impact on population‐level productivity is more limited. Our findings may also be biased toward those studies reporting successful measures, though negative effects of AES have proved highly publishable and an inversed bias may be as likely. Also, we emphasize that our estimation of the probability of success of interventions is likely conservative, as we rated “success” as an intervention having a significant positive effect; thus, studies with insufficient statistical power to detect a significant effect will rate as “unsuccessful” in our analysis, but may potentially have a positive effect. This conservative assessment may contribute to our finding that while most conservation measures are more successful than expected by chance alone, most are unlikely to succeed more than 50% of the time. With more studies reporting effect sizes, a formal meta‐analysis could in future provide an estimate of overall effect size on breeding waders for the interventions we evaluated, overcoming this limitation of our analysis.

Finally, a large proportion of studies examined count metrics, which could be particularly biased by non‐random use of interventions with respect to spatial patterns in wader occurrence or density, an issue highlighted by Kleijn and Sutherland (2003). We therefore suggest that our findings examining trend and productivity metrics are likely the most insightful, although accept that without appropriate study design or statistical controls, studies evaluating these metrics may also potentially be subject to a degree of spatial bias. However, our analysis shows that the success rate for count metrics was not consistently greater than other metrics across all interventions (Figure 3), suggesting that our results are not simply a function of the non‐random distribution of interventions. This adds confidence to our analysis of overall effects (Analysis 1), variation between species (Analysis 2a) and the analysis of interventions applied in combination (Analysis 3), which are based on results combined across different metrics.

## 5 CONCLUSION AND MANAGEMENT IMPLICATIONS

Our assessment suggests that policy and management measures already in place in many European countries to conserve grassland‐breeding waders in intensive agricultural landscapes are broadly effective, although success rate may vary substantially depending on context and the interventions used. In particular, we advocate the use of measures that improve productivity, which is likely to be driving wader population declines and limiting recovery. Our results indicate that AES and site protection are likely key policy tools for improving wader productivity and population trends, while important management interventions are likely to include a reduction in mowing, careful application of light grazing, reduced use of agrochemicals, and increasing wet conditions, though their success is likely to be context‐dependent. Where predation limits breeding success, nest protection, preferably fencing, and/or predator control are recommended, although success may depend on the specific context as well as on the use of the above measures which also improve habitat condition. More studies investigating the ability of interventions to improve chick survival are required since this may be a key factor limiting populations despite positive outcomes for nest survival.

While our assessment shows that conservation measures are more successful at achieving positive outcomes than expected by chance, wader populations continue to decline. This suggests that success rates may not be as high as they need to be, that the magnitude of positive effects may be too small (see Supporting Information Table S3 for summary of effect size ranges), and/or the scale at which they are applied is unable to compensate for declines occurring outside managed areas. Restoration of sustainable wader populations in the wider countryside will depend on the implementation of effective measures at a much greater scale to increase the amount of land managed favorably for these and other grassland species (Vickery & Tayleur, 2018; Walker et al., 2018). This will be governed by the level of support and funding provided by Europe‐wide institutions and national governments, which ultimately will depend on sufficient social will to drive the changes needed to protect and restore farmland biodiversity. The future status of grassland‐breeding waders across Europe will to a large extent depend on further developments in agricultural practices and policy. These are important messages at a time of uncertainty for national and European institutions.

## CONFLICT OF INTEREST

The authors declare no conflict of interest exists.

## AUTHOR CONTRIBUTIONS

SF, MR, WT, and JPH conceived the ideas and designed methodology; SF, MR, and AC extracted the data; SF and MR analyzed the data; SF, MR, and JPH led the writing of the manuscript. All authors contributed critically to the drafts and gave final approval for publication.

## DATA ACCESSIBILITY

The code and data used in the analysis are available from Zenodo https://doi.org/10.5281/zenodo.1419185.
