# Supplementary material for: Evaluating the effectiveness of conservation measures for European grassland‐breeding waders
Source: Ecol Evol. 2018 Oct 9;8(21):10555–68. doi: 10.1002/ece3.4532 (PMC6238142; doi:10.1002/ece3.4532)
Supplement: Supplementary file 1 [file ECE3-8-10555-s001.docx]

# Supplementary Information

## Appendix S1: Search terms used for primary literature review

The following keyword and logic terms were used to search ISI Web of Knowledge on 12 November 2015: (meadowbird OR meadow-bird* OR wader* OR shorebird* OR (lapwing* OR “Vanellus vanellus”) OR (oystercatcher* OR “Haematopus ostralegus”) OR (redshank* OR “Tringa totanus”) OR (ruff OR ruffs OR “Philomachus pugnax”) OR (snipe* OR “Gallinago gallinago”) OR (curlew* OR “Numenius arquata”) OR (dunlin* OR “Calidris alpina”) OR (black-tailed godwit* OR “Limosa limosa”) OR farmland-bird* OR grassland-bird*) in relation to conservation management interventions (conservation OR management OR protect* OR restor* OR reserve OR agri-environment OR graz* OR mow* OR predat* OR fenc* OR drain* OR water OR wet* OR flood* OR agricultur* OR fertiliz* OR fertilis* OR disturb* OR forest* OR tree* OR wood* OR farm* OR arable OR livestock OR cattle OR upland* OR grassland* OR meadow* OR coast* OR (open* SAME landscape) OR till* OR plough* OR sward). The search was constrained to the Web of Science Core Collection and the Scientific Citation Index Expanded (SCI-EXPANDED index) for the years 1980-2015. The search was refined to include only English language results.

## Appendix S2: Methods for identifying grey literature studies

We employed a range of methods to identify relevant grey literature studies, using experts in the field, searching national ornithological journals, and searching the websites of ornithological organisations. First, we contacted experts from four countries (Denmark, Estonia, Czech Republic, Germany) and asked them to provide grey literature material and the names of additional experts. We browsed the websites of 22 national ornithological journals (Table S1) from ten European countries and one international journal which is not ISI-listed (Wader Study, formerly Wader Study Group Bulletin). Additionally, Dutch ([www.natuurtijdschriften.nl/natuur](http://www.natuurtijdschriften.nl/natuur)) and German ([www.ornithologische-schriftenschau.de](http://www.ornithologische-schriftenschau.de)) journals were searched using keywords. Furthermore, we searched the literature databases of two ornithological organisations in the Netherlands (Sovon Dutch Centre for Field Ornithology) and the UK (RSPB), using species names, ‘meadow birds’, ‘management’, ‘protection’ and ‘evaluation’, and specific author names and countries. We searched for reports on the websites of seven national ornithological organisations (research institutes, NGOs and Birdlife International partners) in five countries known to have worked on meadow birds, and searched both Google Scholar and ResearchGate for reports and articles of known meadow bird researchers. We also checked literature cited in both scientific and grey literature that we had already found for further references. We only included grey literature in our meta-analysis if statistical tests were used to quantify the results.

## Appendix S3: Data extraction and synthesis

### Variables extracted

SF (primary literature) and MR (grey literature) extracted the following data for each study (Table S2): location (country, region), start and end year, duration (number of years), the primary and secondary habitat types, the type of study (e.g experimental, semi-experimental or correlative), the type of analysis (e.g. univariate or multivariate), the study’s sample size (small, n < 30; medium, n = 30-100; large, n > 100), which interventions were evaluated, the species and metric(s) for which effects were assessed, and the significance, direction, and magnitude of the effect (where provided) on the metric of interest with (e.g. after/treatment) and without (e.g. before/control) intervention(s). Where possible, effect sizes were either extracted from the text of the Results section, from tables, or from figures using a free, web-based tool (WebPlotDigitizer, <http://arohatgi.info/WebPlotDigitizer/>).

### Studies testing the effect of interventions on multiple metrics and/or species simultaneously

Where a study investigated the effect of interventions on multiple metrics and/or species simultaneously, we extracted each metric and species combination as a separate record. For example, a study which evaluated the simultaneous impacts of reducing mowing frequency and increasing the amount of surface water on abundance and nest survival of black-tailed godwit and lapwing would comprise four individual lines of data in the database: one for the impact of the interventions on black-tailed godwit abundance, one for the impact of the interventions on black-tailed godwit nest survival, one for the impact of the interventions on lapwing abundance, and one for the impact of the interventions on lapwing nest survival.

### Pooling forms of nest protection, agrochemicals and water management

We considered separating nest protection against predation from nest protection against agricultural activities in the original analysis, and we extracted data in such a way as to differentiate between these two different forms of protection. However, we opted to combine them for several reasons. From a practical perspective, we had problems with model convergence due to too few studies using one or the other form of protection when assessed independently. Due to our need to simplify the number of different forms of management interventions for the analysis, we considered pooling nest protection against predation with predator control. However, we deemed that nest protection against predation was more similar in the nature of the techniques used to nest protection against agricultural activities – namely, nests must be found and marked and protected in some way. Furthermore, nests protected for one purpose (e.g. predation) may also serve to protect nests for another purpose (against agricultural damage). However, we do acknowledge that nest protection against agricultural activities may in fact increase predation rates (Kragten, Nagel & De Snoo 2008; Kentie *et al.* 2015; but see Zámečník, Kubelka & Šálek 2018).

Similarly, in order to avoid problems with model convergence due to insufficient sample size, we needed to pool the use of fertilisers (both animal manure and artificial) and herbicides/pesticides, though we extracted these data separately. For the same reason, we also pooled groundwater (e.g. water table manipulation) and surface water management (e.g. footdrains, scrapes and pools) into a single water management category.

### Assessment of study quality

Study quality comprised a qualitative, combined assessment by SF and MR (cross-checked for consistency) of the study’s scientific design and analytical rigour. High quality studies could be either experimental (either as treatment-control or before-after experiments), semi-experimental (e.g. ‘natural experiments’ such as quantitative observational comparisons between two different groups in a case-control design), or quantitative correlative analyses; but they also had to use a robust analytical framework, controlling for confounding variables and problems such as lack of data independence (e.g. through the use of mixed effects models). Medium quality studies could include any of the above scientific designs, though were more likely to be semi-experimental or correlative and use a moderately robust analytical framework (e.g. control for confounding variables but not use a mixed effects model framework). Poor quality studies tended to be correlative or descriptive, and failed to account for potential confounding variables or lack of data independence.

# Tables

Table S1. The non-ISI-listed national or international journals searched for relevant grey literature.

| **Journal Title** | **Country** |
| --- | --- |
| Wader Study (Group Bulletin) | International |
| Notatki Ornitologiczne/ Ornis Polonica | Poland |
| Kulon | Poland |
| Ptaki Polski | Poland |
| Ptaki Pomorza | Poland |
| Sylvia | Czech Republic |
| Alauda | France |
| Dansk Ornitologisk Forenings Tidsskrift | Denmark |
| Die Vogelwelt | Germany |
| Vogelwarte | Germany |
| Charadrius | Germany |
| Corax | Germany |
| Acta Zoologica Lituanica | Lithuania |
| Hirundo | Estonia |
| Ornis Hungarica | Hungary |
| Twirre | Netherlands |
| Vanellus | Netherlands |
| Het Vogeljaar | Netherlands |
| De Levende Natuur | Netherlands |
| Limosa | Netherlands |
| Landschap | Netherlands |
| Aves | Belgium |
| Le Gerfaut | Belgium |

Table S2. Detailed information on the variables used in 1 ) the analysis of the effect of confounding covariates on study success rate; and 2) the analyses (1-3) of success and failure rates of interventions. For interventions, detailed information on the types of specific activities comprised is given. Application of an intervention is relative to a baseline reference level which is less than the level that is applied. Reduction of an intervention is relative to a baseline reference level that is greater than the level to which the intervention has been reduced.

| **Variable type** | **Variable** | **Variable levels (categorical)** | **Details** |
| --- | --- | --- | --- |
| Confounding covariate | Study duration |  | Number of years study was conducted for. |
|  | Sample size | small (< 30) | Sample size used to calculate the effect size of an intervention on a metric. |
|  |  | medium (30-100) |  |
|  |  | large (100-1000) |  |
|  |  | very large (1000-10000) |  |
|  | Analysis type | univariate | Modelling approach used by a study. |
|  |  | multivariate |  |
|  | Literature type | primary | Whether study was primary or grey literature. |
|  |  | grey |  |
|  | Study quality | low | A combined assessment of a study's scientific design and analytical rigour (Appendix S3). |
|  |  | medium |  |
|  |  | high |  |
|  | Metric bias | Yes | Whether a record evaluated the effect on a count-type metric (abundance/occupancy). Count-type metrics could be biased by non-random use of interventions with respect to spatial patterns in wader occurrence or density (Kleijn & Sutherland 2003). Sites for AES are often selected on the basis of occupancy and/or densities of focal species. For some schemes, presence and/or minimum densities, but not trends or productivity, are a prerequisite. Trends and productivity are therefore less likely to be biased, though these may also be affected by initial densities. |
|  |  | No |  |
| Fixed (Analysis 2, 3) or random (Analysis 1) effect covariate | Species | black-tailed godwit | Species evaluated by a study. Records for ruff were excluded as there were too few for the analysis. |
|  |  | curlew |  |
|  |  | dunlin |  |
|  |  | lapwing |  |
|  |  | oystercatcher |  |
|  |  | redshank |  |
|  |  | snipe |  |
| Fixed (Analysis 2) effect covariate | Metric | Abundance/occupancy | Metric evaluated by a study. Records for adult survival and recruitment were excluded as there were too few for the analysis. Abundance/occupancy includes breeding densities and occupancy probability. |
|  |  | Abundance/occupancy change | Includes change in population per year or over a time period, change in occupancy. |
|  |  | Productivity | Includes nest survival, chick survival, nest and chick survival combined, and fledglings per pair. |
| Response | Success | 1/0 | 1 = outcome of intervention(s) evaluated is significantly positive; 0 = outcome of intervention(s) evaluated is not significant or is significantly negative. |
|  | Failure | 1/0 | 1 = outcome of intervention(s) evaluated is significantly negative; 0 = outcome of intervention(s) evaluated is not significant or is significantly positive. |
| **Intervention type** | **Intervention** | **Intervention levels** | **Range of specific activities** |
| Policy | AES | basic | Can be applied at either the site (field/pasture) or landscape (multiple sites) scale. Comprises generic 'biodiversity-friendly' measures not targeted at any specific species. Scheme application, extent, and management measures may vary (Fig. S1). |
|  |  | higher | Can be applied at either the site (field/pasture) or landscape (multiple sites) scale. Comprises measures targeted at outcomes for breeding grassland waders. Scheme application, extent, and management measures may vary (Fig. S1). |
|  | Site protection | applied | Includes nature reserves, national (e.g. Site of Special Scientific Interest in the UK) or international (e.g. Natura 2000) designations. Management measures employed vary according to the form of site protection and extent of management targeted towards specific species (Fig. S1). |
|  |  | reduced | None |
| Management | Mowing | applied | Mowing for hay is applied at a site-specific (field/meadow) or landscape (multiple sites) scale. |
|  |  | reduced | Mowing is restricted at either the site (field/meadow) or landscape (multiple sites) scale. Restrictions may include any of: delaying the start of mowing until after the nesting or chick-rearing season; reduced driving speed during mowing to allow chicks to escape. |
|  | Grazing | applied | Grazing is applied at some level at the site (field/pasture) or landscape (multiple sites) scale. Application is relative to a baseline reference level that is lower than that used. Grazing may be at intensive (high stocking density) or extensive (low stocking density) levels; grazing may be applied early or later in spring relative to baseline. |
|  |  | reduced | Grazing is restricted at some level at the site (field/pasture) or landscape (multiple sites) scale. Restriction is relative to a baseline reference level that is greater than that used. Restrictions may include any of: reducing stocking densities during the nesting and chick-rearing season; delaying the start of grazing until after the nesting or chick-rearing season; cessation of grazing during the breeding season. |
|  | Agrochemicals | applied | Agrochemicals (fertilisers/herbicides/pesticides) are employed. Use may include: spread of fertiliser artificial or manure; use of herbicides/pesticides. In practice, application of agrochemicals as a management tool always referred to fertilisers. |
|  |  | reduced | Application of agrochemicals (fertilisers/herbicides/pesticides) is restricted. Restrictions may include any of: restricted, patchwise instead of field-scale application of herbicides; restriction on amounts or prohibition of fertiliser (artificial or manure) application; restriction on amounts or prohibition of herbicide or pesticide application; organic farming. |
|  | Wet conditions | applied | Wet conditions are increased at a site (field/pasture) or landscape (multiple sites) scale. Availability may be increased by any of: inhibiting groundwater drainage or applying water in any way to increase overall water levels; increasing the amount/extent of (flooded) footdrains; creating scrapes and pools which flood with surface water. |
|  |  | reduced | Wet conditions are restricted at a site- (field/pasture) scale. Restrictions include activities facilitating drainage or removal of surface water. |
|  | Nest protection | applied | Nest protection is applied. Forms may include protection against agricultural activities: marking nests with sticks and leaving unmown patches of variable size around nests; placing rough grids over individual nests to protect against trampling. Forms may also include protection against nest predation: placing cages over individual nests; placing fences around individual nests; placing fences around large areas to protect multiple nests and chicks. Fences could provide either simple physical barriers to exclude mammals of varying size, or could be electrified. |
|  |  | reduced | None |
|  | Predator control | applied | Predator control is applied. Forms may include lethal control (for any of foxes, corvids, mustelids, occasionally raptors or gulls); removal and translocation (hedgehogs). |
|  |  | reduced | None |

Table S3. Effect sizes could be extracted for 48 of the studies reviewed, resulting in 286 records. Minimum and maximum standardised effect sizes, summarised across all studies, are shown for all species combined for abundance/occupancy, abundance/occupancy change, and productivity metrics. Standardised effect sizes were calculated as *(smi – smc)/smi*, where *smi* = the standardised metric with the intervention, and *smc* = the standardised metric without the intervention. The sample size refers to the number of individual records in the dataset that report an effect size for the given intervention. The reported effect size of an intervention is not necessarily independent of other interventions that are applied simultaneously for a given record, as some studies controlled for the combined application of interventions, while others did not.

|  |  | **Abundance/occupancy** | | | **Abundance/occupancy change** | | | **Productivity** | | |
| --- | --- | --- | --- | --- | --- | --- | --- | --- | --- | --- |
| **Management intervention evaluated** | | **min** | **max** | **n** | **min** | **max** | **n** | **min** | **max** | **n** |
| AES | overall | -1.00 | 20.00 | 70 | -1.00 | 0.14 | 38 | -0.02 | 2.43 | 19 |
|  | basic | -1.00 | 9.50 | 49 | -1.00 | 0.06 | 13 | 0.18 | 2.43 | 5 |
|  | higher | 0.00 | 20.00 | 21 | -0.13 | 0.14 | 25 | -0.02 | 1.33 | 14 |
| site protection |  | -0.80 | 32.75 | 40 | -1.00 | 0.16 | 26 | 0.02 | 1.80 | 8 |
| mowing | applied |  |  |  | 0.03 | 0.07 | 5 | -0.50 | 0.25 | 6 |
|  | reduced | -0.53 | 2.30 | 16 | -0.13 | 0.12 | 12 | -0.59 | 1.80 | 9 |
| grazing | applied | -0.78 | 1.38 | 12 | 0.03 | 0.07 | 5 | -0.81 | 4.00 | 17 |
|  | reduced | -0.59 | 2.30 | 11 |  |  |  | 0.00 | 1.21 | 5 |
| agrochemicals | applied |  |  |  | 0.03 | 0.07 | 5 | -0.62 | 0.12 | 8 |
|  | reduced | 0.29 | 1.00 | 5 |  |  |  | 0.32 | 1.33 | 3 |
| water | applied | -0.38 | 2.68 | 18 | 0.03 | 0.07 | 5 | -0.69 | 2.00 | 19 |
|  | reduced | 0.58 | 0.58 | 1 |  |  |  | -0.62 | 1.80 | 7 |
| nest protection | overall | -0.31 | 6.70 | 15 | -0.13 | 0.12 | 12 | -0.59 | 38.02 | 36 |
|  | from agriculture | -0.31 | 6.70 | 15 | -0.13 | 0.12 | 12 | -0.59 | 8.24 | 19 |
|  | from predation |  |  |  |  |  |  | -0.24 | 38.02 | 18 |
| predator control | applied | -0.67 | 37.00 | 8 | -0.01 | 1.59 | 3 | 0.86 | 3.14 | 6 |

Table S4. Model structure for each analysis (1-3) with formulae given in R notation and the R function used to run the model. The given data categories removed were to prevent model singularity or complete/quasi-complete separation.

| **Analysis** | **Intervention** | **Intervention levels** | **Model** | **R function** | **Data categories removed to prevent model singularity or separation** |
| --- | --- | --- | --- | --- | --- |
| 1a | AES | applied | success = 1 + (1\|study) + (1\|species), family=binomial | glmer |  |
|  | AES level | basic, higher | success = AES level + (1\|study) + (1\|species), family=binomial | glmer |  |
|  | site protection | applied | success = 1 + (1\|study) + (1\|species), family=binomial | glmer |  |
|  | mowing | applied, reduced | success = mowing + (1\|study) + (1\|species), family=binomial | glmer |  |
|  | grazing | applied, reduced | success = grazing + (1\|study) + (1\|species), family=binomial | glmer |  |
|  | agrochemicals | applied, reduced | success = agrochemicals + (1\|study) + (1\|species), family=binomial | glmer |  |
|  | water availability | applied, reduced | success = water + (1\|study) + (1\|species), family=binomial | glmer |  |
|  | nest protection | applied | success = 1 + (1\|study) + (1\|species), family=binomial | glmer |  |
|  | predator control | applied | success = 1 + (1\|study) + (1\|species), family=binomial | glmer |  |
| 1b | AES | applied | failure = 1 + (1\|study) + (1\|species), family=binomial | glmer |  |
|  | AES level | basic, higher | failure = AES level + (1\|study) + (1\|species), family=binomial | glmer |  |
|  | site protection | applied | failure = 1 + (1\|study) + (1\|species), family=binomial | glmer |  |
|  | mowing | applied, reduced | failure = mowing + (1\|study) + (1\|species), family=binomial | glmer |  |
|  | grazing | applied, reduced | failure = grazing + (1\|study) + (1\|species), family=binomial | glmer |  |
|  | agrochemicals | applied, reduced | failure = agrochemicals + (1\|study) + (1\|species), family=binomial | glmer |  |
|  | water availability | applied, reduced | failure = water + (1\|study) + (1\|species), family=binomial | glmer |  |
|  | nest protection | applied | failure = 1 + (1\|study) + (1\|species), family=binomial | glmer |  |
|  | predator control |  | Unsolvable model convergence problems |  |  |
| 2a | AES | applied | success = species + (1\|study), family=binomial | bglmer | dunlin |
|  | AES level | basic, higher | success = AES level + species + AES level*species + (1\|study), family=binomial | bglmer | dunlin |
|  | site protection | applied | success = species + (1\|study), family=binomial | bglmer | dunlin |
|  | mowing | reduced | success = species + (1\|study), family=binomial | bglmer | dunlin, curlew; mowing applied |
|  | grazing | applied, reduced | success = grazing + species + grazing*species + (1\|study), family=binomial | bglmer | dunlin, curlew |
|  | agrochemicals | reduced | success = species + (1\|study), family=binomial | bglmer | curlew, dunlin, oystercatcher; redshank, snipe; agrochemicals applied |
|  | water availability | applied | success = species + (1\|study), family=binomial | bglmer | dunlin, curlew, snipe |
|  | nest protection | applied | success = species + (1\|study), family=binomial | bglmer | curlew |
|  | predator control | applied | success = species + (1\|study), family=binomial | bglmer | snipe, oystercatcher, redshank |
| 2b | AES | applied | success = metric + (1\|study), family=binomial | bglmer |  |
|  | AES level | basic, higher | success = AES level + metric + AES level*metric + (1\|study), family=binomial | bglmer |  |
|  | site protection | applied | success = metric + (1\|study), family=binomial | bglmer |  |
|  | mowing | applied, reduced | success = mowing + metric + mowing*metric + (1\|study), family=binomial | bglmer |  |
|  | grazing | applied, reduced | success = grazing + metric + grazing*metric + (1\|study), family=binomial | bglmer | abundance/occupancy change |
|  | agrochemicals | reduced | success = metric + (1\|study), family=binomial | bglmer | agrochemicals applied |
|  | water availability | applied, reduced | success = water + metric + water*metric + (1\|study), family=binomial | bglmer | abundance/occupancy change |
|  | nest protection | applied | success = metric + (1\|study), family=binomial | bglmer |  |
|  | predator control | applied | success = metric + (1\|study), family=binomial | bglmer | abundance/occupancy |
| 3a | Policy | AES-no site protection, no AES-site protection, AES-site protection | success = Policy + species + (1\|study), family=binomial | glmer |  |
| 3b | Management |  | success = mowing + grazing + agrochemicals + water + nest protection + predator control + species + (1\|study), family=binomial | glmer |  |

Table S5. Likelihood ratio test results for confounding covariates removed using single-term deletion from the model *logit(π_i_) = α+β_1_ x study duration_i_+β_2_ x sample size_i_+β_3_ x analysis type_i_+β_4_ x literature type_i_+β_5_ x study quality_i_+β_6_ x metric bias_i_+a_1i_+ε_i_,* where π_i_=success and study (a_1_) is fitted as a random intercept.

| **Variable** | **df** | **χ^2^** | **p** |
| --- | --- | --- | --- |
| study duration | 1 | 1.561 | 0.211 |
| sample size | 2 | 0.596 | 0.742 |
| analysis type | 2 | 4.073 | 0.130 |
| literature type | 1 | **6.498** | **0.011** |
| study quality | 2 | 4.067 | 0.131 |
| metric bias | 1 | 0.054 | 0.816 |

Table S6. Mean predicted probability ± 95% confidence interval that the given intervention level will result in a significant positive (Analysis 1a, 2ab, 3) or negative (Analysis 1b) outcome for the species or metric given.

| **Analysis** | **Management intervention evaluated** | | **Species or Metric** | **Mean predicted probability of success** | **95% confidence interval** |
| --- | --- | --- | --- | --- | --- |
| 1a) Success | AES |  |  | 0.312 | 0.19, 0.467 |
|  | site protection |  |  | 0.476 | 0.318, 0.639 |
|  | AES level | basic |  | 0.206 | 0.114, 0.342 |
|  |  | higher |  | 0.473 | 0.303, 0.651 |
|  | mowing | applied |  | 0.154 | 0.021, 0.61 |
|  |  | reduced |  | 0.261 | 0.123, 0.471 |
|  | grazing | applied |  | 0.289 | 0.169, 0.449 |
|  |  | reduced |  | 0.244 | 0.094, 0.5 |
|  | agrochemicals | applied |  | 0.027 | 0, 0.84 |
|  |  | reduced |  | 0.644 | 0.108, 0.964 |
|  | water | applied |  | 0.420 | 0.177, 0.708 |
|  |  | reduced |  | 0.127 | 0.016, 0.57 |
|  | nest protection |  |  | 0.357 | 0.238, 0.496 |
|  | predator control |  |  | 0.133 | 0.006, 0.785 |
| 1b) Failure | AES | basic |  | 0.050 | 0.029, 0.084 |
|  | site protection | higher |  | 0.059 | 0.023, 0.144 |
|  | AES level | applied |  | 0.016 | 0.002, 0.113 |
|  |  | reduced |  | 0.006 | 0, 0.586 |
|  | mowing | applied |  | 0.102 | 0.012, 0.527 |
|  |  | reduced |  | 0.047 | 0.01, 0.2 |
|  | grazing | applied |  | 0.129 | 0.048, 0.302 |
|  |  | reduced |  | 0.017 | 0.001, 0.183 |
|  | agrochemicals | applied |  | 0.119 | 0.005, 0.786 |
|  |  | reduced |  | 0.043 | 0.002, 0.544 |
|  | water |  |  | 0.009 | 0, 0.166 |
|  |  |  |  | 0.172 | 0.012, 0.779 |
|  | nest protection |  |  | 0.001 | 0, 0.358 |
| 2a) Species | AES |  | black-tailed godwit | 0.399 | 0.214, 0.619 |
|  |  |  | curlew | 0.130 | 0.038, 0.36 |
|  |  |  | lapwing | 0.361 | 0.218, 0.533 |
|  |  |  | oystercatcher | 0.154 | 0.047, 0.402 |
|  |  |  | redshank | 0.401 | 0.232, 0.597 |
|  |  |  | snipe | 0.293 | 0.134, 0.526 |
|  | site protection |  | black-tailed godwit | 0.537 | 0.286, 0.771 |
|  |  |  | curlew | 0.090 | 0.015, 0.385 |
|  |  |  | lapwing | 0.485 | 0.288, 0.687 |
|  |  |  | oystercatcher | 0.584 | 0.258, 0.85 |
|  |  |  | redshank | 0.547 | 0.349, 0.731 |
|  |  |  | snipe | 0.441 | 0.234, 0.672 |
|  | AES level | basic | black-tailed godwit | 0.226 | 0.085, 0.479 |
|  |  |  | curlew | 0.113 | 0.026, 0.377 |
|  |  |  | lapwing | 0.198 | 0.094, 0.369 |
|  |  |  | oystercatcher | 0.126 | 0.029, 0.414 |
|  |  |  | redshank | 0.324 | 0.163, 0.541 |
|  |  |  | snipe | 0.204 | 0.071, 0.463 |
|  |  | higher | black-tailed godwit | 0.149 | 0.31, 0.813 |
|  |  |  | curlew | 0.609 | 0.023, 0.568 |
|  |  |  | lapwing | 0.176 | 0.381, 0.797 |
|  |  |  | oystercatcher | 0.486 | 0.027, 0.621 |
|  |  |  | redshank | 0.412 | 0.25, 0.728 |
|  |  |  | snipe | 0.382 | 0.172, 0.703 |
|  | mowing | reduced | black-tailed godwit | 0.382 | 0.182, 0.632 |
|  |  |  | lapwing | 0.223 | 0.064, 0.549 |
|  |  |  | oystercatcher | 0.097 | 0.015, 0.433 |
|  |  |  | redshank | 0.164 | 0.038, 0.498 |
|  |  |  | snipe | 0.238 | 0.029, 0.765 |
|  | grazing | applied | black-tailed godwit | 0.247 | 0.067, 0.6 |
|  |  |  | lapwing | 0.376 | 0.174, 0.632 |
|  |  |  | oystercatcher | 0.272 | 0.077, 0.624 |
|  |  |  | redshank | 0.224 | 0.076, 0.503 |
|  |  |  | snipe | 0.197 | 0.025, 0.702 |
|  | grazing | reduced | black-tailed godwit | 0.301 | 0.06, 0.743 |
|  |  |  | lapwing | 0.341 | 0.078, 0.759 |
|  |  |  | oystercatcher | 0.199 | 0.021, 0.739 |
|  |  |  | redshank | 0.172 | 0.019, 0.687 |
|  |  |  | snipe | 0.202 | 0.022, 0.744 |
|  | agrochemicals | reduced | black-tailed godwit | 0.779 | 0.255, 0.973 |
|  |  |  | lapwing | 0.474 | 0.07, 0.915 |
|  | water | applied | black-tailed godwit | 0.685 | 0.39, 0.881 |
|  |  |  | lapwing | 0.389 | 0.15, 0.697 |
|  |  |  | oystercatcher | 0.174 | 0.027, 0.617 |
|  |  |  | redshank | 0.431 | 0.165, 0.744 |
|  | nest protection |  | black-tailed godwit | 0.242 | 0.085, 0.524 |
|  |  |  | dunlin | 0.275 | 0.039, 0.779 |
|  |  |  | lapwing | 0.456 | 0.251, 0.678 |
|  |  |  | oystercatcher | 0.126 | 0.018, 0.536 |
|  |  |  | redshank | 0.441 | 0.173, 0.748 |
|  |  |  | snipe | 0.313 | 0.037, 0.845 |
|  | predator control |  | curlew | 0.135 | 0.001, 0.973 |
|  |  |  | lapwing | 0.063 | 0, 0.958 |
| 2b) Metric | AES |  | abundance/occupancy | 0.257 | 0.153, 0.399 |
|  |  |  | abundance/occupancy change | 0.302 | 0.176, 0.466 |
|  |  |  | productivity | 0.550 | 0.292, 0.784 |
|  | site protection |  | abundance/occupancy | 0.429 | 0.256, 0.622 |
|  |  |  | abundance/occupancy change | 0.626 | 0.439, 0.782 |
|  |  |  | productivity | 0.333 | 0.092, 0.711 |
|  | AES level | basic | abundance/occupancy | 0.189 | 0.098, 0.333 |
|  |  |  | abundance/occupancy change | 0.157 | 0.069, 0.318 |
|  |  |  | productivity | 0.411 | 0.085, 0.841 |
|  |  | higher | abundance/occupancy | 0.310 | 0.156, 0.522 |
|  |  |  | abundance/occupancy change | 0.617 | 0.361, 0.821 |
|  |  |  | productivity | 0.629 | 0.326, 0.857 |
|  | mowing | applied | abundance/occupancy | 0.137 | 0.014, 0.635 |
|  |  |  | abundance/occupancy change | 0.656 | 0.153, 0.953 |
|  |  |  | productivity | 0.053 | 0.004, 0.465 |
|  |  | reduced | abundance/occupancy | 0.154 | 0.05, 0.386 |
|  |  |  | abundance/occupancy change | 0.199 | 0.057, 0.507 |
|  |  |  | productivity | 0.573 | 0.241, 0.85 |
|  | grazing | applied | abundance/occupancy | 0.296 | 0.13, 0.541 |
|  |  |  | productivity | 0.136 | 0.033, 0.423 |
|  |  | reduced | abundance/occupancy | 0.218 | 0.057, 0.564 |
|  |  |  | productivity | 0.295 | 0.042, 0.8 |
|  | agrochemicals | reduced | abundance/occupancy | 0.850 | 0.055, 0.998 |
|  |  |  | abundance/occupancy change | 0.305 | 0.002, 0.988 |
|  |  |  | productivity | 0.956 | 0.037, 1 |
|  | water | applied | abundance/occupancy | 0.448 | 0.161, 0.775 |
|  |  |  | productivity | 0.388 | 0.104, 0.776 |
|  |  | reduced | abundance/occupancy | 0.237 | 0.015, 0.862 |
|  |  |  | productivity | 0.092 | 0.004, 0.74 |
|  | nest protection |  | abundance/occupancy | 0.209 | 0.061, 0.516 |
|  |  |  | abundance/occupancy change | 0.350 | 0.097, 0.729 |
|  |  |  | productivity | 0.447 | 0.277, 0.631 |
|  | predator control |  | abundance/occupancy change | 0.116 | 0.004, 0.796 |
|  |  |  | productivity | 0.278 | 0.015, 0.905 |
| 3) | mowing | applied |  | 0.157 | 0.02, 0.63 |
|  |  | reduced |  | 0.285 | 0.074, 0.66 |
|  | grazing | applied |  | 0.297 | 0.08, 0.701 |
|  |  | reduced |  | 0.295 | 0.071, 0.676 |
|  | agrochemicals | applied |  | 0.136 | 0.016, 0.605 |
|  |  | reduced |  | 0.447 | 0.133, 0.803 |
|  | water | applied |  | 0.382 | 0.106, 0.767 |
|  |  | reduced |  | 0.177 | 0.025, 0.643 |
|  | nest protection |  |  | 0.373 | 0.116, 0.723 |
|  | predator control |  |  | 0.318 | 0.077, 0.704 |

Table S7. Likelihood ratio test results for the analysis of management interventions applied in combination and removing each covariate using single-term deletion from the model *logit(π_i_) = α+β_1_ x mowing_i_+β_2_ x grazing_i_+β_3_ x agrochemicals_i_+β_4_ x water_i_+β_5_ x nest protection_i_+β_6_ x predator control_i_+β_7_ x Species_i_+a_1i_+ε_i_* where π_i_=success and study (a_1_) is fitted as a random intercept.

| **Variable** | **df** | **χ^2^** | **p** |
| --- | --- | --- | --- |
| mowing | 2 | 1.116 | 0.572 |
| grazing | 2 | 0.537 | 0.765 |
| agrochemicals | 2 | 2.743 | 0.254 |
| water | 2 | 3.107 | 0.211 |
| nest protection | 1 | 1.240 | 0.265 |
| predator control | 1 | 0.007 | 0.934 |

Table S8. The grey and primary literature references, each arranged alphabetically by surname of first author, included in the dataset for the meta-analysis. A total of 74 studies were used for the meta-analysis, 58 from the primary literature and 16 from the grey literature.

| **Literature type** | **Reference** |
| --- | --- |
| grey | Berg, A. et al. 1994.; Lapwings Vanellus vanellus nesting on farmland - can the farmer save the nests? |
| grey | Boschert, M. 2008. Protection Measures for Curlew Clutches – Experiences of electric fencing in the Upper Rhine Valley. Naturschutz und Landschaftsplanung 40 (10): 346-352. |
| grey | Bruns, H.A., H. Hötker, J. Christiansen, B. Hälterlein,& W. Petersen-Andresen, 2001. Brutbestände und Bruterfolg von Wiesenvögeln im Beltringharder Koog (Nordfriesland) in Abhängigkeit von Sukzession, Beweidung, Wasserständen und Prädatoren. Corax 18, Sonderheft 2: 67-80 |
| grey | Clausen, P. & J. Kahlert, 2010. YNGLEFUGLE I TØNDERMARSKEN OG MARGRETHE KOG 1975-2009. En analyse af udviklingen i fuglenes antal og fordeling med anbefalinger til forvaltningstiltag. Faglig rapport fra DMU nr. 778 |
| grey | Kentie, R., Hooijmeijer, J.C.E.W., Both, C. & Piersma, T. 2011. Grutto's in ruimte en tijd 2007-2010, eindrapport |
| grey | Molenaar, J.G. de, D.A. Jonkers, P. Vereijken en G. Kolkman, 2005. EHS-Experiment Gaasterland; 2.Effectiviteit Agrarisch Weidevogelbeheer. Wageningen, Alterra, Alterra-rapport 1131. |
| grey | Nijland, F., 2007. Een succesvol broedjaar voor weidevogels in 2006 |
| grey | Olsen, H., 2003. Impacts of electric fence exclosures on Lapwing Vanellus vanellus breeding success. In: Patterns of predation on ground nesting meadow birds, PhD thesis, the Royal Veterinary and Agricultural University Copenhagen, Denmark |
| grey | Oosterveld E.B. m.m.v. Sovon Vogelonderzoek Nederland 2015. Effecten van opkrikmaatregelen in Friese weidevogelreservaten op de weidevogels 2007-2013. A&Wrapport 2133. Altenburg & Wymenga ecologisch onderzoek, Feanwâlden |
| grey | Schifferli, L. , R. Spaar & A. Koller. 2006. Fence and plough for Lapwings: Nest protection to improve nest and chick survival in Swiss farmland. Osnabrücker Naturwissenschaftliche Mitteilungen 32 S 123-129 |
| grey | Stanbury, A., O'Brien, M., & A. Donaghy, 2000. Trends in breeding wader populations in key areas within Northern Ireland between 1986 and 2000. Irish Birds 6: 513-526 |
| grey | Struwe-Juhl, B. 1995. Auswirkungen der Renaturierungsmassnamen im Hohner See-Gebiet auf bestand, Bruterfolg und Nahrungsökolgie der Uferschnepfe (Limosa limosa). Corax 16:153-171 |
| grey | Teunissen, W.A. & Willems, F. 2004. Bescherming van weidevogels |
| grey | Thorup, O., 2004. Status of populations and management of Dunlin *Calidris alpina*, Ruff *Philomachus pugnax* and Black-tailed Godwit *Limosa limosa* in Denmark. Dansk Orntihologisk Forening Tidsskrift 98: 21-32 |
| grey | Van Egmond & de Koeijer, 2006, Weidevogelbeheer bij agrariërs en terreinbeheerders |
| primary | Amar, A. et al. 2011. Exploring the relationships between wader declines and current land-use in the British uplands. - Bird Study 58: 13–26. |
| primary | Amar, A., Thirgood, S., Pearce-Higgins, J. & Redpath, S. (2008) The impact of raptors on the abundance of upland passerines and waders. Oikos, 117, 1143–1152. |
| primary | Armsworth, P.R., Acs, S., Dallimer, M., Gaston, K.J., Hanley, N. & Wilson, P. (2012) The cost of policy simplification in conservation incentive programs. Ecology Letters, 15, 406–414. |
| primary | Ausden, M. & Hirons, G.J.M. (2002) Grassland nature reserves for breeding wading birds in England and the implications for the ESA agri-environment scheme. Biological Conservation, 106, 279–291. |
| primary | Baines, D. 1989. The effects of improvement of upland, marginal grasslands on the breeding success of lapwings Vanellus-vanellus and other waders. - Ibis 131: 497–506. |
| primary | Baines, D. 1990. The roles of predation, food and agricultural practice in determining the breeding success of the lapwing (Vanellus-vanellus) on upland grasslands. - Journal of Animal Ecology 59: 915–929. |
| primary | Baines, D. et al. 2008. The direct and indirect effects of predation by Hen Harriers Circus cyaneus on trends in breeding birds on a Scottish grouse moor. - Ibis 150: 27–36. |
| primary | Baldi, A. et al. 2005. Effects of grazing intensity on bird assemblages and populations of Hungarian grasslands. - Agriculture Ecosystems & Environment 108: 251–263. |
| primary | Bellebaum, J. and Bock, C. 2009. Influence of ground predators and water levels on Lapwing Vanellus vanellus breeding success in two continental wetlands. - Journal of Ornithology 150: 221–230. |
| primary | Berg, A. et al. 2002. Population dynamics and reproduction of Northern Lapwings Vanellus vanellus in a meadow restoration area in central Sweden. - Ibis 144: E131–E140. |
| primary | Bodey, T.W., Mcdonald, R.A., Sheldon, R.D. & Bearhop, S. (2011) Absence of effects of predator control on nesting success of Northern Lapwings Vanellus vanellus: implications for conservation. Ibis, 153, 543–555. |
| primary | Bolton, M., Tyler, G., Smith, K. & Bamford, R. (2007) The impact of predator control on lapwing Vanellus vanellus breeding success on wet grassland nature reserves. Journal of Applied Ecology, 44, 534–544. |
| primary | Bradbury, R.B. & Allen, D.S. (2003) Evaluation of the impact of the pilot UK Arable Stewardship Scheme on breeding and wintering birds. Bird Study, 50, 131–141. |
| primary | Bright, J.A., Morris, A.J., Field, R.H., Cooke, A.I., Grice, P.V., Walker, L.K., Fern, J. & Peach, W.J. (2015) Higher-tier agri-environment scheme enhances breeding densities of some priority farmland birds in England. Agriculture Ecosystems & Environment, 203, 69–79. |
| primary | Broyer, J. et al. 2014. How to improve agri-environment schemes to achieve meadow bird conservation in Europe? A case study in the Saone valley, France. - Journal of Ornithology 155: 145–155. |
| primary | Calladine, J. et al. 2014. Conservation management of moorland: a case study of the effectiveness of a combined suite of management prescriptions which aim to enhance breeding bird populations. - Bird Study 61: 56–72. |
| primary | Chamberlain, D. et al. 2009. Bird use of cultivated fallow “Lapwing plots” within English agri-environment schemes. - Bird Study 56: 289–297. |
| primary | Davey, C. M. et al. 2010. Assessing the impact of Entry Level Stewardship on lowland farmland birds in England. - Ibis 152: 459–474. |
| primary | Douglas, D. J. T. et al. 2014. Upland land use predicts population decline in a globally near-threatened wader. - Journal of Applied Ecology 51: 194–203. |
| primary | Durant, D. et al. 2008. Field occupancy by breeding lapwings Vanellus vanellus and redshanks Tringa totanus in agricultural wet grasslands. - Agriculture Ecosystems & Environment 128: 146–150. |
| primary | Eglington, S. M. et al. 2008. Restoration of wet features for breeding waders on lowland grassland. - Journal of Applied Ecology 45: 305–314. |
| primary | Fletcher, K., Aebischer, N.J., Baines, D., Foster, R. & Hoodless, A.N. (2010) Changes in breeding success and abundance of ground-nesting moorland birds in relation to the experimental deployment of legal predator control. Journal of Applied Ecology, 47, 263–272. |
| primary | Groen, N.M., Kentie, R., de Goeij, P., Verheijen, B., Hooijmeijer, J.C.E.W. & Piersma, T. (2012) A modern landscape ecology of Black-tailed Godwits: habitat selection in southwest Friesland, The Netherlands. Ardea, 100, 19–28. |
| primary | Hart, J.D., Milsom, T.P., Baxter, A., Kelly, P.F. & Parkin, W.K. (2002) The impact of livestock on Lapwing Vanellus vanellus breeding densities and performance on coastal grazing marsh. Bird Study, 49, 67–78. |
| primary | Henderson, I.G., Holland, J.M., Storkey, J., Lutman, P., Orson, J. & Simper, J. (2012) Effects of the proportion and spatial arrangement of un-cropped land on breeding bird abundance in arable rotations. Journal of Applied Ecology, 49, 883–891. |
| primary | Isaksson, D. et al. 2007. Managing predation on ground-nesting birds: The effectiveness of nest exclosures. - Biological Conservation 136: 136–142. |
| primary | Jackson, D. B. 2001. Experimental removal of introduced hedgehogs improves wader nest success in the Western Isles, Scotland. - Journal of Applied Ecology 38: 802–812. |
| primary | Kahlert, J. et al. 2007. Response of breeding waders to agri-environmental schemes may be obscured by effects of existing hydrology and farming history. - Journal of Ornithology 148: S287–S293. |
| primary | Kentie, R. et al. 2013. Intensified agricultural use of grasslands reduces growth and survival of precocial shorebird chicks. - Journal of Applied Ecology 50: 243–251. |
| primary | Kentie, R. et al. 2015. Management of modern agricultural landscapes increases nest predation rates in Black-tailed Godwits Limosa limosa. - Ibis 157: 614–625. |
| primary | Kragten, S., Nagel, J.C. & De Snoo, G.R. (2008) The effectiveness of volunteer nest protection on the nest success of Northern Lapwings Vanellus vanellus on Dutch arable farms. Ibis, 150, 667–673. |
| primary | Laursen, K. & Hald, A.B. (2012) Identification of Black-tailed Godwit’s Limosa limosa breeding habitat by botanical and environmental indicators. Journal of Ornithology, 153, 1141–1152. |
| primary | Malpas, L.R., Kennerley, R.J., Hirons, G.J.M., Sheldon, R.D., Ausden, M., Gilbert, J.C. & Smart, J. (2013) The use of predator-exclusion fencing as a management tool improves the breeding success of waders on lowland wet grassland. Journal for Nature Conservation, 21, 37–47. |
| primary | Mandema, F. S. et al. 2013. Livestock grazing and trampling of birds’ nests: an experiment using artificial nests. - Journal of Coastal Conservation 17: 409–416. |
| primary | Norris, K. et al. 1998. Is the density of redshank Tringa totanus nesting on saltmarshes in Great Britain declining due to changes in grazing management? - Journal of Applied Ecology 35: 621–634. |
| primary | O’Brien, M. and Wilson, J. D. 2011. Population changes of breeding waders on farmland in relation to agri-environment management. - Bird Study 58: 399–408. |
| primary | Olsen, H. and Schmidt, N. M. 2004. Impacts of wet grassland management and winter severity on wader breeding numbers in eastern Denmark. - Basic and Applied Ecology 5: 203–210. |
| primary | Oosterveld, E. B. et al. 2011. Effectiveness of spatial mosaic management for grassland breeding shorebirds. - Journal of Ornithology 152: 161–170. |
| primary | Ottvall, R. and Smith, H. G. 2006. Effects of an agri-environment scheme on wader populations of coastal meadows of southern Sweden. - Agriculture Ecosystems & Environment 113: 264–271. |
| primary | Pakanen, V.-M. et al. 2011. Nest predation and trampling as management risks in grazed coastal meadows. - Biodiversity and Conservation 20: 2057–2073. |
| primary | Parr, R. (1993). Nest predation and numbers of golden plovers *Pluvialis apricaria* and other moorland waders. Bird Study, 40, 223–231. |
| primary | Pauliny, A., Larsson, M. & Blomqvist, D. (2008) Nest predation management: Effects on reproductive success in endangered shorebirds. Journal of Wildlife Management, 72, 1579–1583. |
| primary | Piha, M., Tiainen, J., Holopainen, J. & Vepsalainen, V. (2007) Effects of land-use and landscape characteristics on avian diversity and abundance in a boreal agricultural landscape with organic and conventional farms. Biological Conservation, 140, 50–61. |
| primary | Rickenbach, O. et al. 2011. Exclusion of ground predators improves Northern Lapwing Vanellus vanellus chick survival. - Ibis 153: 531–542. |
| primary | Schekkerman, H. et al. 2008. The effect of “mosaic management” on the demography of black-tailed godwit Limosa limosa on farmland. - Journal of Applied Ecology 45: 1067–1075. |
| primary | Schekkerman, H., Teunissen, W. & Oosterveld, E. (2009) Mortality of Black-tailed Godwit Limosa limosa and Northern Lapwing Vanellus vanellus chicks in wet grasslands: influence of predation and agriculture. Journal of Ornithology, 150, 133–145. |
| primary | Sheldon, R., Bolton, M., Gillings, S. & Wilson, A. (2004) Conservation management of Lapwing Vanellus vanellus on lowland arable farmland in the UK. Ibis, 146, 41–49. |
| primary | Sheldon, R.D., Chaney, K. & Tyler, G.A. (2007) Factors affecting nest survival of Northern Lapwings Vanellus vanellus in arable farmland: an agri-environment scheme prescription can enhance nest survival. Bird Study, 54, 168–175. |
| primary | Smart, J., Bolton, M., Hunter, F., Quayle, H., Thomas, G. & Gregory, R.D. (2013) Managing uplands for biodiversity: Do agri-environment schemes deliver benefits for breeding lapwing Vanellus vanellus? Journal of Applied Ecology, 50, 794–804. |
| primary | Smart, J., Gill, J.A., Sutherland, W.J. & Watkinson, A.R. (2006) Grassland-breeding waders: identifying key habitat requirements for management. Journal of Applied Ecology, 43, 454–463. |
| primary | Smart, J., Wotton, S.R., Dillon, I.A., Cooke, A.I., Diack, I., Drewitt, A.L., Grice, P.V. & Gregory, R.D. (2014) Synergies between site protection and agri-environment schemes for the conservation of waders on lowland wet grasslands. Ibis, 156, 576–590. |
| primary | Stevens, D.K. & Bradbury, R.B. (2006) Effects of the Arable Stewardship Pilot Scheme on breeding birds at field and farm-scales. Agriculture Ecosystems & Environment, 112, 283–290. |
| primary | Verhulst, J. et al. 2007. Direct and indirect effects of the most widely implemented Dutch agri-environment schemes on breeding waders. - Journal of Applied Ecology 44: 70–80. |
| primary | Verhulst, J., Kleijn, D., Loonen, W., Berendse, F. & Smit, C. (2011) Seasonal distribution of meadow birds in relation to in-field heterogeneity and management. Agriculture Ecosystems & Environment, 142, 161–166. |
| primary | Vickery, J.A., Sutherland, W.J., OBrien, M., Watkinson, A.R. & Yallop, A. (1997) Managing coastal grazing marshes for breeding waders and overwintering geese: Is there a conflict? Biological Conservation, 79, 23–34. |
| primary | Watson, A. & Rae, R. (1997) Some effects of set-aside on breeding birds in northeast Scotland. Bird Study, 44, 245–251. |
| primary | Wilson, A., Vickery, J. & Pendlebury, C. (2007) Agri-environment schemes as a tool for reversing declining populations of grassland waders: Mixed benefits from Environmentally Sensitive Areas in England. Biological Conservation, 136, 128–135. |
| primary | Wilson, A.M., Vickery, J.A., Brown, A., Langston, R.H.W., Smallshire, D., Wotton, S. & Vanhinsbergh, D. (2005) Changes in the numbers of breeding waders on lowland wet grasslands in England and Wales between 1982 and 2002. Bird Study, 52, 55–69. |

# Figures


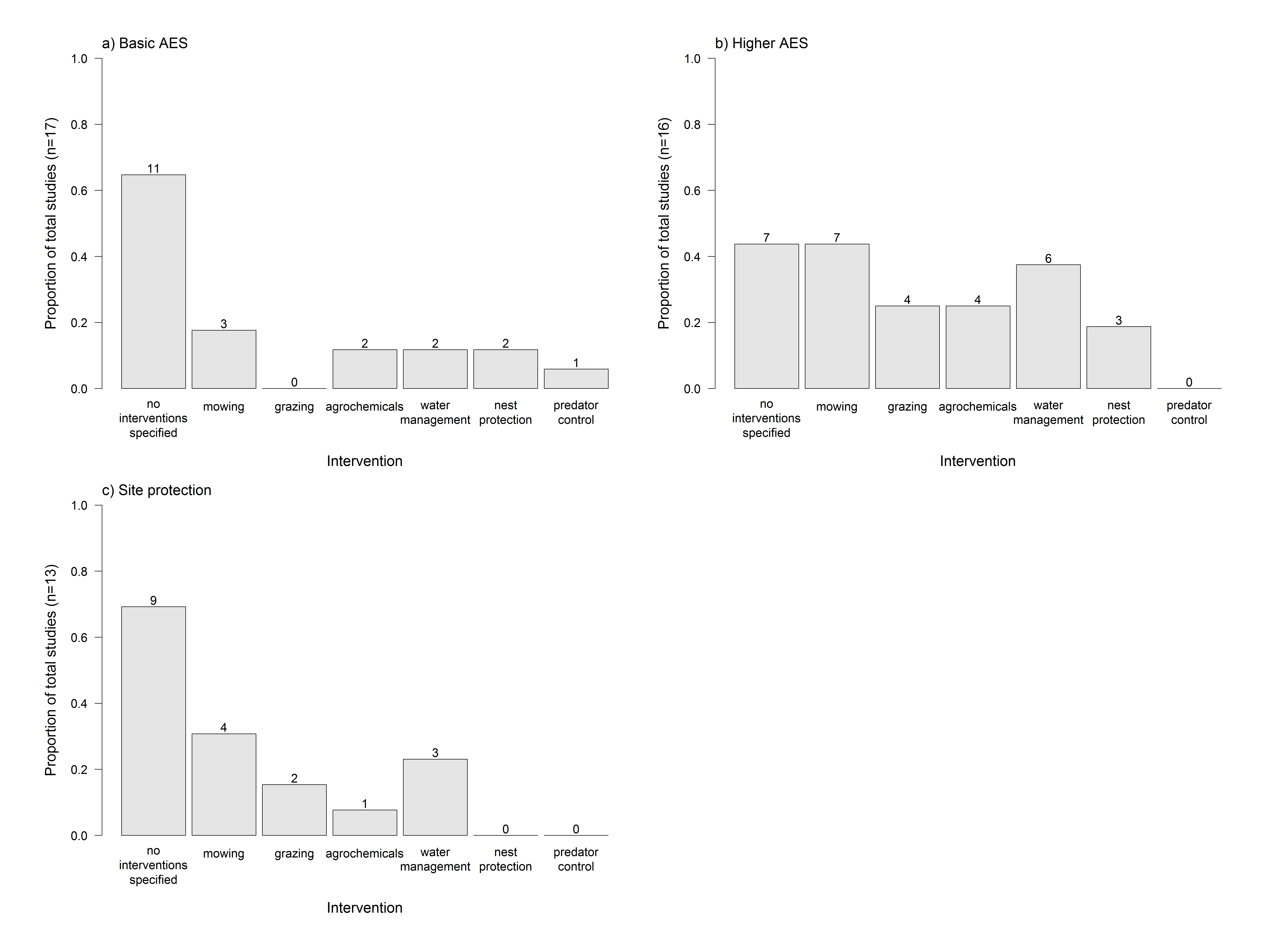


Figure S1. The proportion of studies evaluating different component management interventions for all studies testing the effects of a) basic AES; b) higher-level AES; and c) site protection. The number of studies is given above the bar.


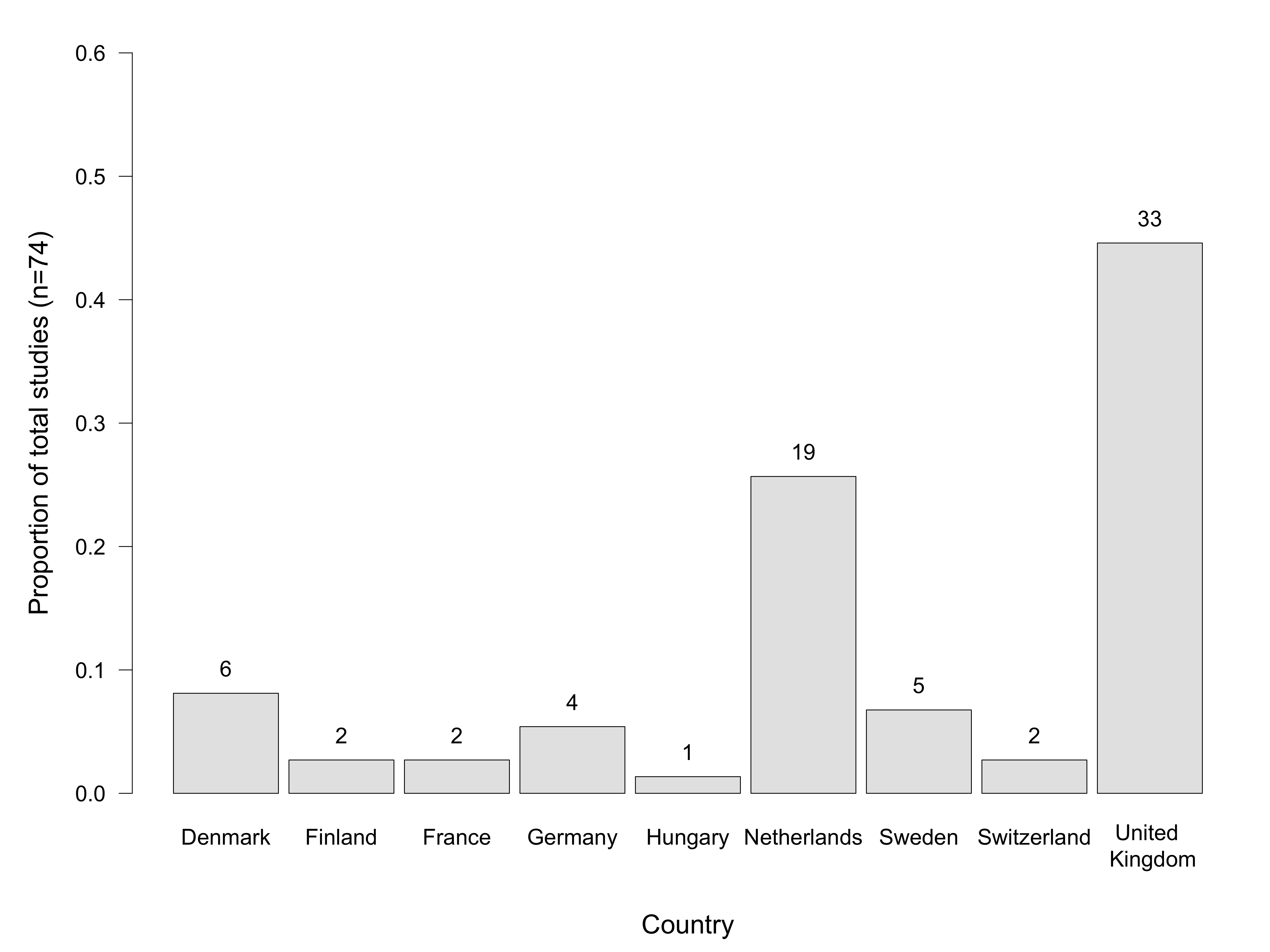


Figure S2. The proportion of the total number of studies which were conducted in different countries. The number of studies from each country is given above the bar.


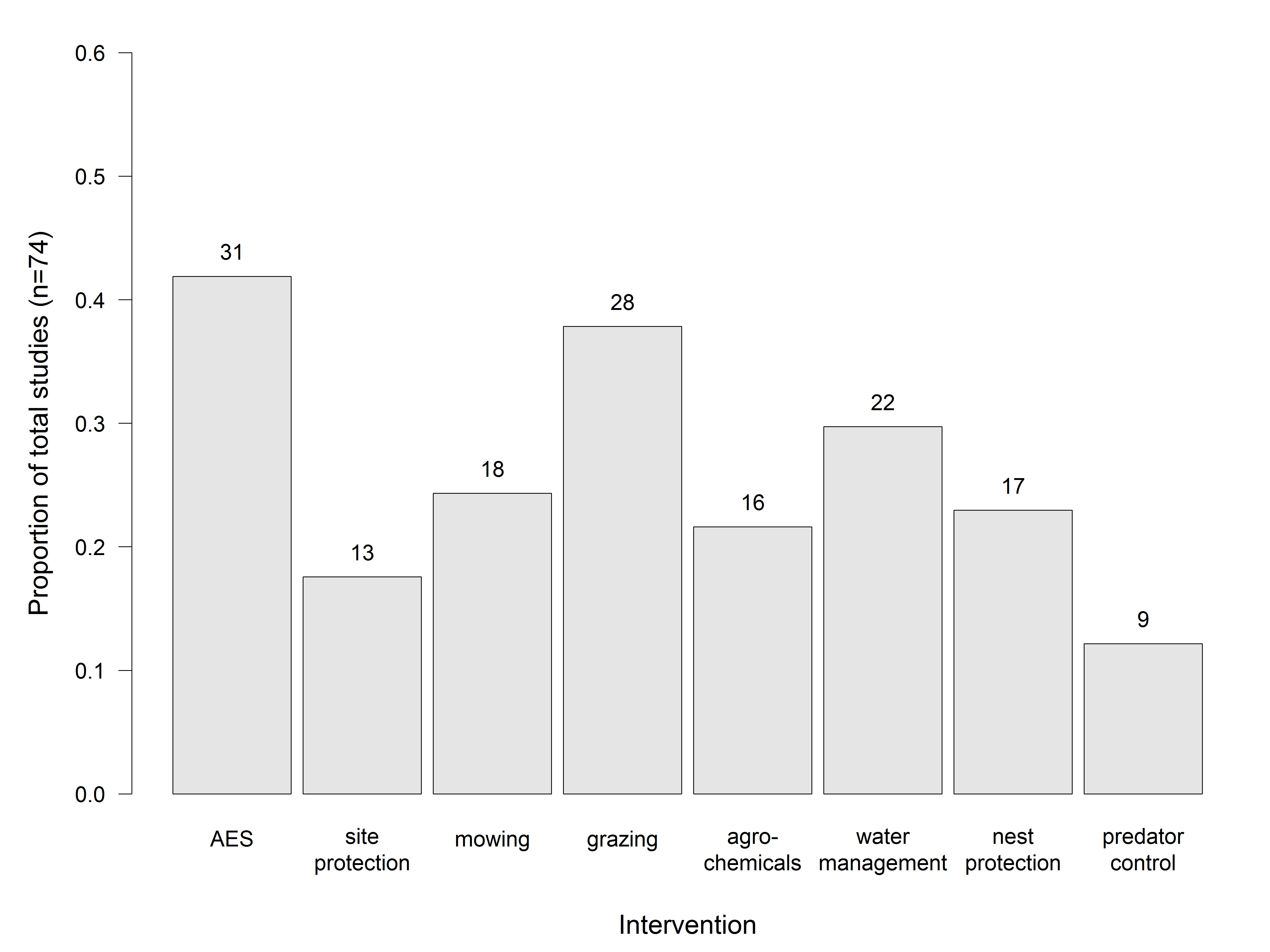


Figure S3. The proportion of the total number of studies which evaluated the impact of a particular policy or management intervention. The number of studies examining each intervention is given above the bar.


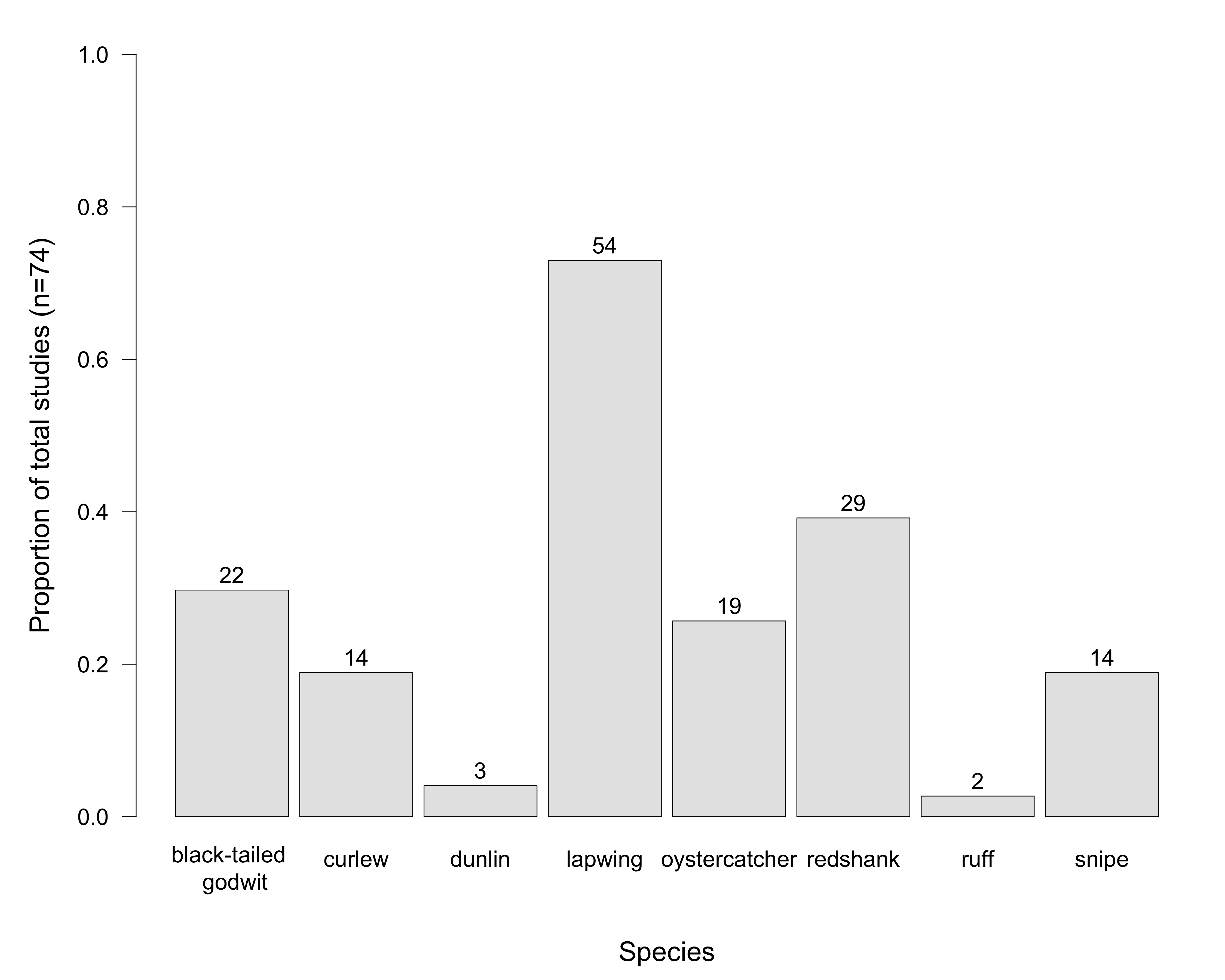


Figure S4. The proportion of the total number of studies which included each species as a study species. The number of studies examining effects on a species are given above the bar.


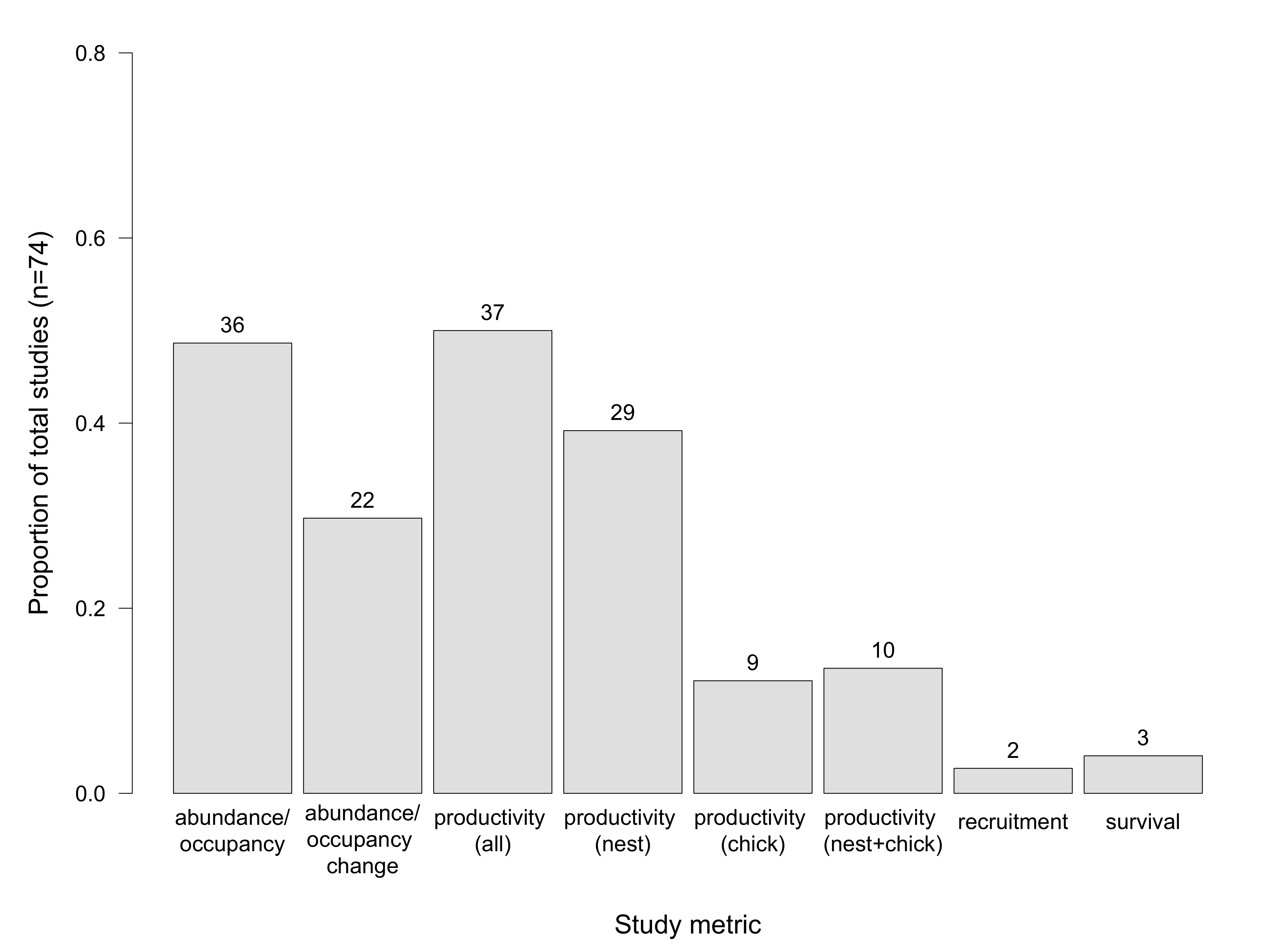


Figure S5. The proportion of the total number of studies which evaluated the impact of management interventions on different population and demographic metrics. The number of studies evaluating a metric is given above the bar.


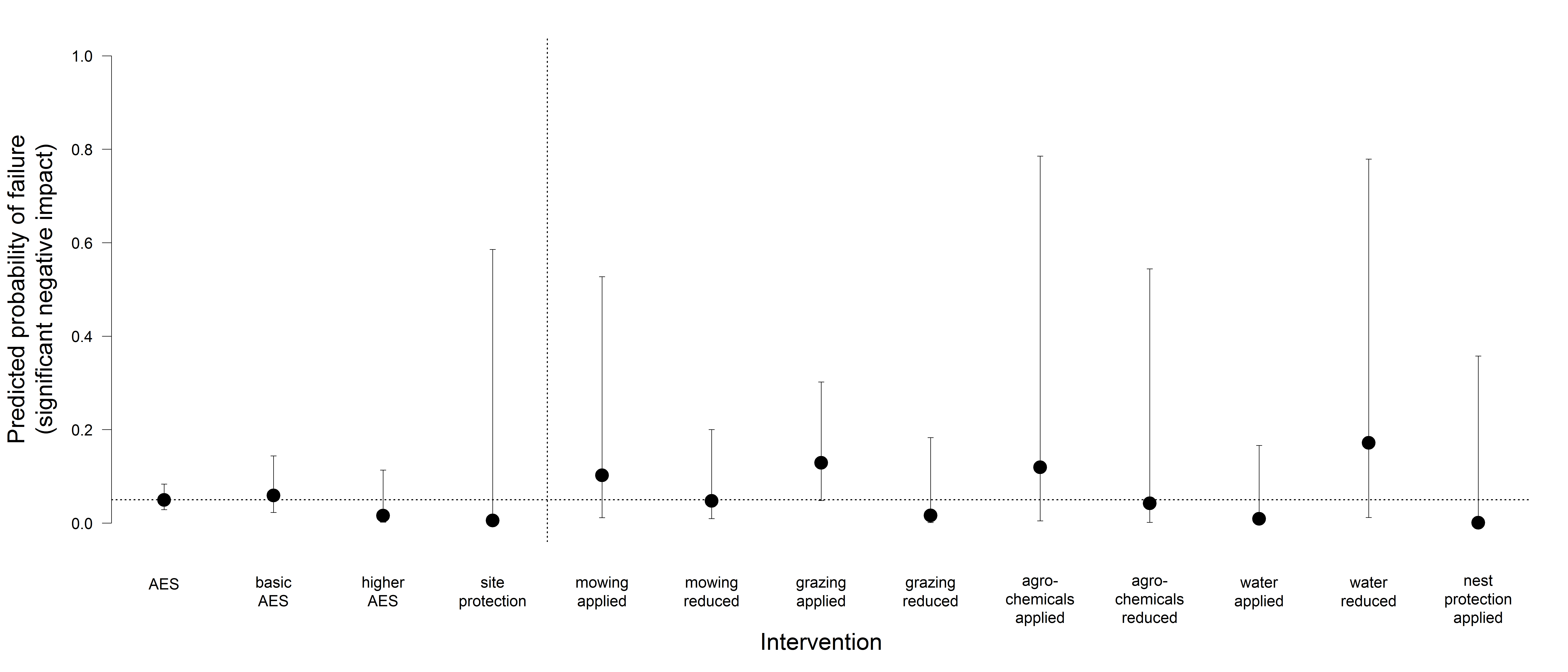


Figure S6. The predicted probability (mean ± 95% confidence interval) that policy and management interventions will result in a significant negative impact (i.e. a significant reduction in any population or demographic metric; Analysis 1b). The dotted horizontal line represents the threshold at which we would expect failure by random chance, at a significance level of p = 0.05. Policy measures are to the left of the vertical dotted line, management measures are to the right. There were too few cases of failed predator control to include this intervention in the model.


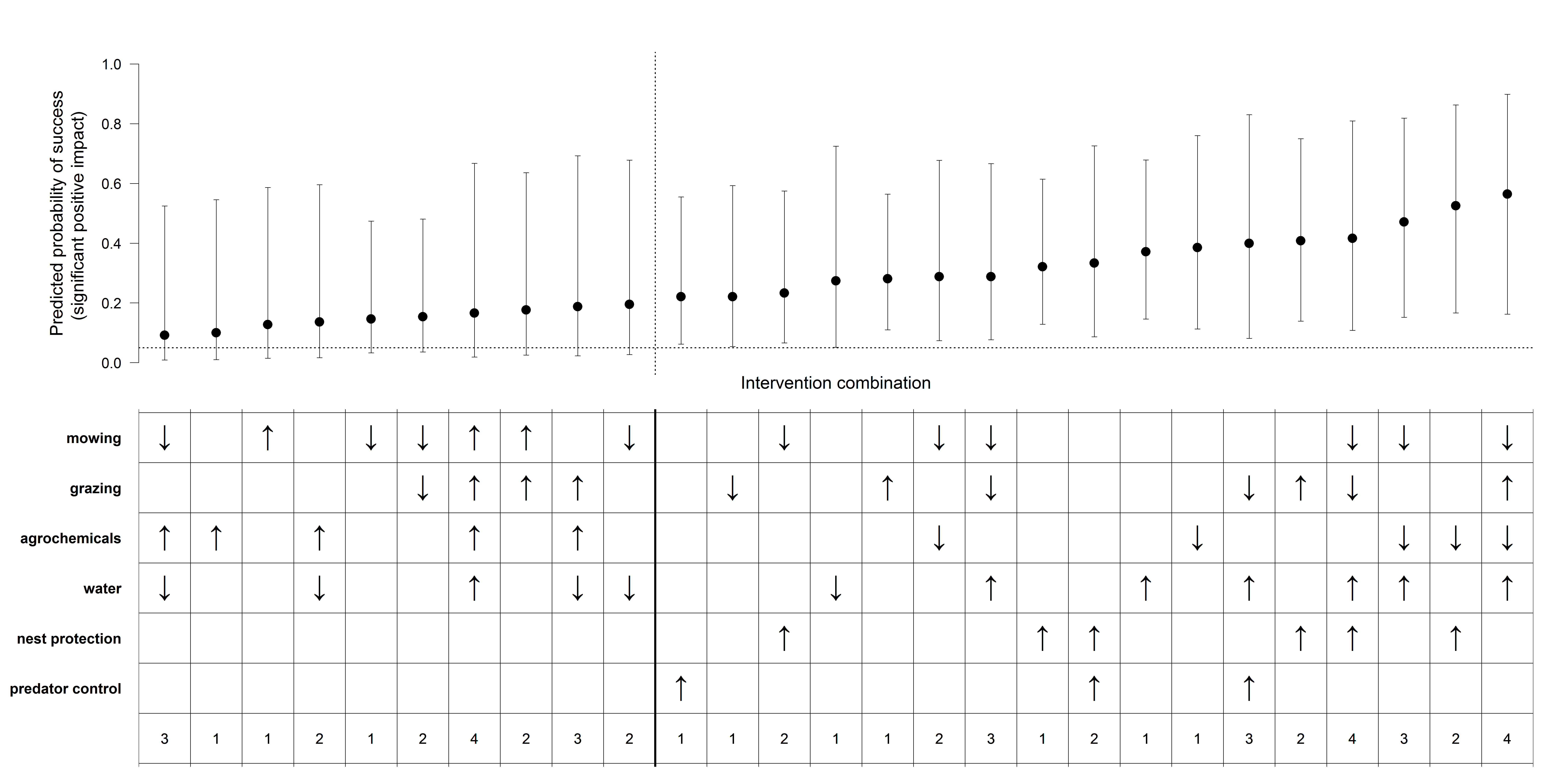


Figure S7. The probability of success (mean ± 95% confidence interval ) for all combinations of interventions employed in the literature we reviewed, ranked according to i) whether the combination has a greater probability of success than expected by chance (95% confidence intervals do not overlap 0.05) and ii) by success rate. The dotted horizontal line represents the 5% threshold for success expected by chance. Interventions to the right of the dotted vertical line and the solid vertical line have a greater chance of success than expected by random chance. The table shows the intervention combination used that contributed to the success rate directly above, with up arrows indicating an intervention was applied and down arrows indicating the intervention was reduced. Numbers on the bottom row show the total number of interventions used in each combination.
